# Contrasting Patterns of rDNA Homogenization within the *Zygosaccharomyces rouxii* Species Complex

**DOI:** 10.1371/journal.pone.0160744

**Published:** 2016-08-08

**Authors:** Tikam Chand Dakal, Paolo Giudici, Lisa Solieri

**Affiliations:** Department of Life Sciences, University of Modena and Reggio Emilia, Via Amendola 2, 42122, Reggio Emilia, Italy; The University of Hong Kong, HONG KONG

## Abstract

Arrays of repetitive ribosomal DNA (rDNA) sequences are generally expected to evolve as a coherent family, where repeats within such a family are more similar to each other than to orthologs in related species. The continuous homogenization of repeats within individual genomes is a recombination process termed concerted evolution. Here, we investigated the extent and the direction of concerted evolution in 43 yeast strains of the *Zygosaccharomyces rouxii* species complex (*Z*. *rouxii*, *Z*. *sapae*, *Z*. *mellis*), by analyzing two portions of the 35S rDNA cistron, namely the D1/D2 domains at the 5’ end of the 26S rRNA gene and the segment including the internal transcribed spacers (ITS) 1 and 2 (ITS regions). We demonstrate that intra-genomic rDNA sequence variation is unusually frequent in this clade and that rDNA arrays in single genomes consist of an intermixing of *Z*. *rouxii*, *Z*. *sapae* and *Z*. *mellis*-like sequences, putatively evolved by reticulate evolutionary events that involved repeated hybridization between lineages. The levels and distribution of sequence polymorphisms vary across rDNA repeats in different individuals, reflecting four patterns of rDNA evolution: I) rDNA repeats that are homogeneous within a genome but are chimeras derived from two parental lineages via recombination: *Z*. *rouxii* in the ITS region and *Z*. *sapae* in the D1/D2 region; II) intra-genomic rDNA repeats that retain polymorphisms only in ITS regions; III) rDNA repeats that vary only in their D1/D2 domains; IV) heterogeneous rDNA arrays that have both polymorphic ITS and D1/D2 regions. We argue that an ongoing process of homogenization following allodiplodization or incomplete lineage sorting gave rise to divergent evolutionary trajectories in different strains, depending upon temporal, structural and functional constraints. We discuss the consequences of these findings for *Zygosaccharomyces* species delineation and, more in general, for yeast barcoding.

## Introduction

**Fig 1:**
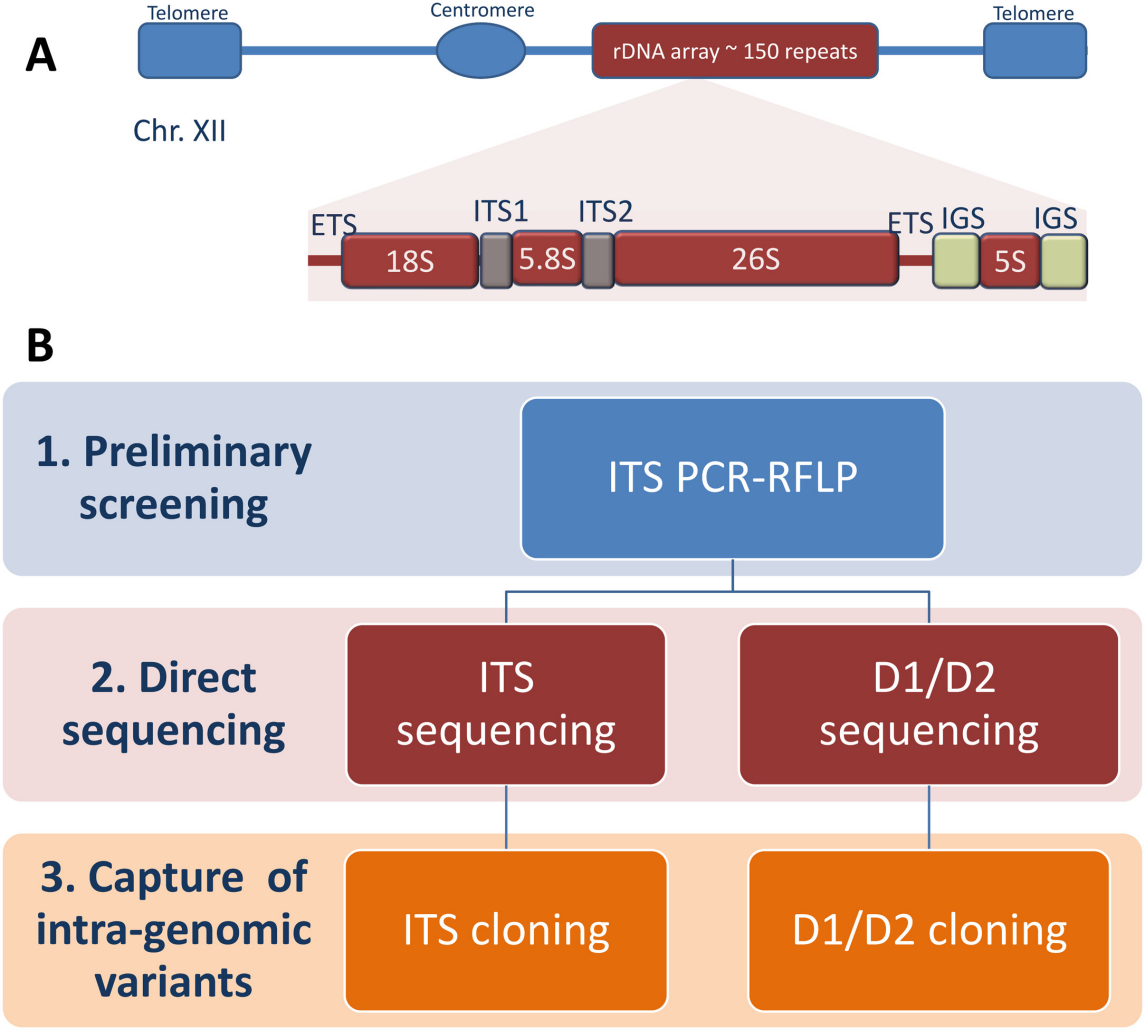
Overview of the experimental design. A) The rDNA locus in chromosome XII of the model yeast *Saccharomyces cerevisiae*, and a schematic representation of its rDNA unit. Boxes and lines represent the rDNA repeat structure and are not to scale. B) A three-step strategy is used to identify intra-genomic variants within a pool of 43 *Z*. *rouxii* related strains. Abbreviations: ETS, external transcribed spacer; ITS, internal transcribed spacer; IGS, intergenic spacer.

Nuclear genes encoding ribosomal RNA (rDNA) are universally distributed across the tree of life and account for more than 50% of total cellular transcripts produced by the cell, depending on organism and growth conditions [1]. To support this level of expression, yeasts and higher eukaryotes possess multicopy nuclear rDNA sequences organized as head-to-tail tandem rDNA arrays in nucleolus organizer regions (NORs). Each tandem array comprises a large precursor 35S RNA consisting of coding sequences for its three subunits, namely the 28S/26S large subunit (LSU), the 18S small subunit, and the 5.8S rRNA genes. These coding genes are separated by two intervening and rapidly evolving non-coding regions, the internal transcribed spacers (ITS1 and ITS2), and together constitute a single transcriptional cistron transcribed by RNA polymerase I [2] (Fig 1A). In most hemiascomycetes, the 5S rRNA gene is present within the array separated from the 35S gene by two non-transcribed intergenic spacers (IGS, also called NTS1 and NTS2) [3]. In yeasts, tandem arrays may contain 45–200 copies of the rDNA repeat unit [4] distributed across one or more chromosomal locations [5, 6].

Because tandem rDNA arrays contain highly conserved genes and variable intergenic regions, they serve as an important molecular clock for inferring evolutionary relationships between organisms [7]. However, the use of rDNA regions as phylogenetic markers would be less reliable if they did not evolve in a concerted fashion. Therefore, it is advisable to ascertain the peculiar mode of rDNA evolution in a genus before its use in phylogenetic study. Like other tandem-repeated gene families, the rDNA repeats do not evolve independently, but in a concerted manner, thanks to continual turnover of repeats wherein new mutations in one gene are either eliminated or spread to adjacent genes, eventually homogenizing all of them. This process, globally referred to as concerted evolution [8, 9], is supposed to homogenize rDNA copies by gene conversion (that is, the copying and pasting of one genomic copy onto another locus, whether they are orthologous or not) and/or unequal crossing over between homologous rDNA units [10–12]. Collectively, the mechanisms of turnover underpin the process of molecular drive, which is the concomitant spread of new variants both through a family (homogenization) and through a sexual species (fixation) with the passing of the generations [12]. Molecular drive gives rise to the observed patterns of within-species homogeneity and between-species diversity among rDNA multigene families [4, 8, 12].

Evidence about the rDNA array concerted evolution has been derived from studies on metazoans (e.g., *Drosophila* and *Xenopus*) [13] and *Saccharomyces cerevisiae* [4]. However, recent studies have shown that several repeat families previously thought to have evolved via concerted evolution, actually evolve according to a birth-and-death mechanism under strong purifying selection [14–17]. This mechanism is characterized by infrequent duplications of repeats, with the initially high level of sequence similarity decaying away through mutations without the action of any homogenizing mechanism. The purifying selection acts to maintain the functional integrity of rDNA copies in spite of their independent evolution from one another. Eickbush and Eickbush [18] presented a comprehensive model encompassing mutation, homologous recombination, and selection as primary forces involved in concerted evolution of the rDNA gene family. In particular, the crossover rate needs to be high compared to the mutation rate to ensure the concerted evolution of rDNA repeats. In case the mutation rate exceeds the crossover rate, significant variations in intra-genomic repeats are expected in regions of loose selective constraints [18]. Accordingly, several reports have demonstrated intra-genomic polymorphisms in the rDNA arrays of prokaryotes [19], plants [20], protists [21, 22], fungi [17], [23–26], and animals [27–30].

Among hemiascomycetes, the *Zygosaccharomyces rouxii* species complex represents a particularly challenging system for phylogenetic reconstruction because it comprises highly variable yeasts, such as the haploid *Z*. *rouxii* species, the diploid sister species *Z*. *sapae* [31, 32], and a subgroup of allodiploid/aneuploid mosaic strains with uncertain taxonomic position and putative hybrid origin [32–34]. These yeasts inhabit food with low water activity (a_w_), and in addition to a marked variation in ploidy level and genome size [35], they also exhibit near-continuity of stress-related phenotypic characters [36], duplication of nuclear genes [33, 35, 37], and a variable degree of rDNA heterogeneity [31–34]. With respect to intra-individual rDNA sequences, *Z*. *sapae* displays homogenized 26S rDNA sequences coupled to variable ITS regions (comprising the highly variable ITS1 and ITS2 as well as the more conserved 5.8S rDNA in between), [32], but the allodiploid/aneuploid mosaic strains possess heterogeneous rDNA arrays with polymorphisms in both the 26S rDNA and the ITS regions [31, 34]. Therefore, the *Z*. *rouxii* complex could be an interesting model system to study mechanisms underlying the rDNA homogenization in yeast. However, no works have explored the occurrence of rDNA heterogeneity in wild-type and collection strains formally ascribed to the species *Z*. *rouxii*. This work aims at filling this gap and at characterizing the extent of rDNA heterogeneity in 43 strains isolated from salty and sugary foodstuffs.

## Materials and Methods

### Yeast strains and experimental design

*Zygosaccharomyces* strains used in this work are listed in S1 Table. According to the procedure described by Solieri et al. [37], 29 strains were isolated from highly sugary cooked musts used in traditional balsamic vinegar (TBV) processing, whereas 19 from spoiled honey samples. Fourteen strains were retrieved from salty food (shoyu mash, shoyu moromi, and miso) and deposited in the Nite Biological Resource Center (NBRC, Japan) under the species name *Z*. *rouxii* (S1 Table). The following strains were used as references for comparative purposes: *Z*. *rouxii* CBS 732^T^, the aneuploid strains CBS 4837 and CBS 4838, *Zygosaccharomyces mellis* CBS 736^T^ and *Zygosaccharomyces bailii* CBS 680^T^ were purchased from the Centraalbureau voor Schimmelcultures (CBS; Utrecht, The Netherlands); *Zygosaccharomyces pseudorouxii* (nom. inval.) NCYC 3042 from the National Collection of Yeast Culture (NCYC; UK); and allodiploid ATCC 42981 from the American Type Culture Collection (ATCC; Rockville, USA). All strains were single-colony cultures obtained after two rounds of routine streaking and were maintained on yeast extract 1% (w/v), peptone 1% (w/v), dextrose 2% (w/v) (YPD) agar plates at 26°C. One millilitre stocks of single-colony cultures containing 25% glycerol (v/v) as cryopreservative were stored at -80°C.

The experimental plan described in Fig 1B was designed to assign a proper taxonomic and phylogenetic position to the new isolates and to identify intra-individual rDNA sequence variation. The experimental plan included three steps: 1) rudimentary assessment of ITS sequence variation by RFLP analysis; 2) direct DNA sequencing of ITS regions and D1/D2 domains; 3) cloning and sequencing of intra-genomic DNA sequence variants, when the direct sequencing obtained for a single-colony culture failed or the electropherograms exhibited polymorphisms.

### PCR-RFLP, cloning and sequencing of rDNA marker

Cells originating from a single colony were inoculated in 4 ml of YPD medium, grown overnight at 26°C, and used for genomic DNA (gDNA) extraction as previously described [38]. Quantity and purity (OD_260/280_ and OD_260/230_) of DNA samples were evaluated using a NanoDrop ND-1000 spectrophotometer. PCR amplification of ITS regions was performed with the primer pair ITS1/ITS4 [39], whereas PCR amplification of 26S rDNA D1/D2 domains was carried out with the primer pair NL-1/NL-4 [40]. All PCR reactions were carried out with high-fidelity *ExTaq* DNA polymerase (Takara, Japan) in a Bio-Rad T100 thermal cycler (Bio-Rad, Laboratories, Hercules, CA), as previously described [31, 32]. To detect ITS intra-genomic polymorphisms, ITS amplicons were subjected to restriction fragment length polymorphism (RFLP) analysis using endonucleases *Hae*III, *Hha*I and *Hinf*I, as previously reported [37] (Fig 1B). Those strains that showed complex ITS profiles (sum of individual bands > size of ITS amplicon) were considered heterogeneous in that ITS region. Based on the ITS-RFLP profile, each strain was assigned a group. One representative strain from each group and the singleton strains (that did not fall in any group based on their unique ITS profile) were subjected to ITS sequencing and 26S D1/D2 PCR and sequencing.

ITS and D1/D2 PCR amplicons were purified using a PCR purification kit (DNA Clean & Concentrator^™^ 25 kit, Zymo Research) and sequenced with the same primers used in the PCR reactions. Where appropriate, we used the internal primers ITS2/ITS3 [39] and NL-2/NL-3 [40], respectively (Fig 1B). Sequences were edited and assembled using Lasergen SeqMan software (DNASTAR, Madison, WI, USA). The absence or presence of notable intra-individual heterogeneity was assessed by visual examination of electropherograms obtained from direct sequencing. The presence of heterogeneities was indicated by double peaks in substitution positions, and by unreadable electropherograms due to a series of mixed peaks in case of indel events, both positioned after a sequence of good quality.

In case of heterogeneity, ITS and D1/D2 PCR amplicons were cloned into the pGEM-T Easy Vector following the manufacturer’s instructions (Promega, Madison, WI). At least fifteen bacterial colonies were randomly selected from the transformants. The plasmids were extracted from the bacterial clones and checked for the size of ITS and D1/D2 inserts by re-amplification with the primer pairs ITS1/ITS4 and NL-1/NL-4, respectively. ITS and D1/D2 inserts were screened by *Hae*III- and *Ava*I-RFLPs. These enzymes were chosen based on the fact that their restriction sites are altered among heterogeneous rDNA copies, and therefore, the use of these discriminatory restriction enzymes leads to confirmation of intra- and inter-strain heterogeneous rDNA copies. At least two representative inserts for each restriction profile were sequenced in both directions as reported above. All the sequencing reactions were carried out by a custom sequencing service provider (BMR Genomics, Padova, Italy). The sequences were deposited in GenBank under accession numbers listed in S2 Table.

### Sequence analysis and RNA secondary structure prediction

All sequences obtained by direct sequencing and/or cloning were BLASTed against the GenBank and YeastIP [41] databases to retrieve sequences of their closest relatives. The sequences were aligned using the ClustalW2 algorithm [42]. ITS1 and ITS2 spacers were identified according to nucleotide coordinates reported by James et al. [43]. D1 and D2 domains were delimited by the NL1 and NL4 primer target sites. Two variable regions inside D1 and D2 domains, (termed VR1 and VR2, respectively) were found from nucleotide position 85 to 194 and from nucleotide position 431 to 525 (numbering refers to *Z*. *rouxii* CBS 732^T^ sequence AY046112), respectively. When required, ITS and D1/D2 sequences were subjected to *in silico* restriction digestion with the commercially available type-II restriction endonucleases listed in the REBASE database (http://rebase.neb.com) [44] by using the web-tool NEBcutter, version 2.0 (http://tools.neb.com/NEBcutter).

Secondary (2D) structures were determined for novel ITS and D1/D2 sequence variants no previously found in other strains. RNA transcript folding was generated separately for ITS1 and ITS2 with RNAfold web-tool (rna.tbi.univie.ac.at/cgi-bin/RNAfold.cgi). The resulting 2D structures were compared to those of *Z*. *rouxii* CBS 732^T^ (GenBank accession number AM279465), *Z*. *sapae* ABT301^T^ (GenBank accession numbers AM279464 to AM279466) and strain NCYC 3042 (GenBank accession number HE984156). The stem-loop structures present in the D1 and D2 domain sequences were predicted with the minimum MFE (maximum stability) using RNA Structure Ver 5.7 (http://rna.urmc.rochester.edu/RNAstructureWeb/). The generated 2D structures were then compared with those of the corresponding parts of *S*. *cerevisiae* (also available at http://www.rna.icmb.utexas.edu/.), *Z*. *rouxii* (GenBank accession number AY046112), *Z*. *sapae* (GenBank accession number AJ966517) and *Z*. *mellis* (GenBank accession number U72164) 26S rRNA molecules.

### Tree-based phylogeny and network analysis

The phylogenetic relationships were reconstructed with the neighbour-joining (NJ) method [45] using ITS (GenBank accession number AY046191) and D1/D2 (GenBank accession number U72161) sequences of *Z*. *bailii* CBS 680^T^ as outgroups. Percentages of replicate trees in which the associated taxa clustered together in the bootstrap test (10,000 replicates) are shown next to the branches in both trees (only values higher than 60% are reported) [46]. Evolutionary divergences between ITS sequences were computed using the Tamura-Nei method [47], while the evolutionary divergences between 26S rDNA D1/D2 sequences were computed using the Tajima-Nei method [48]. The DNA substitution models that best fitted both our datasets were chosen with the software jModelTest 2.1.7 [49]. All these analyses were carried out in MEGA6 [50].

Network analysis was performed using the SplitsTree4 V4.12.8 package [51], taking as input the ClustalW2 alignments of ITS and D1/D2 sequences. For distance calculation, the distance estimation method K3ST (Kimura’s three-substitution types) was used. The Neighbor-net algorithm was used to draw an unrooted split-network, and the Equal Angle setting was chosen. The Phi-test for recombination as implemented in SplitsTree4 program was performed. As the split network based on the Neighbor-Net algorithm often gives conflicting signals, we computed conflicting signals across taxa using delta scores and Q-residual scores, as available in the SplitsTree4 program.

## Results

### Analysis of ITS RLFP data

ITS restriction fingerprinting was generated by three endonucleases on a panel of 43 strains isolated from salty and sugary food and 9 reference yeasts, as summarized in Table 1. ITS restriction patterns sorted out 43 strains in 9 groups or genotypes (from G-1 to G-9), whereas 9 strains remained singletons (clusters with a single strain that showed unique ITS restriction profiles). Out of 19 strains isolated from spoiled honey samples, 17 showed genotypes G-1 to G-3, while strain 70 showed an atypical G-2 profile due to a restriction site gain/loss for the endonuclease *Hha*I. Genotype G-1 grouped 7 honey strains and *Z*. *mellis* CBS 736^T^, whereas genotype G-2 grouped the remaining 7 honey strains but did not match any genotype of the reference strains included in this work. Previous works demonstrated that *Z*. *mellis* comprises two types, namely α and β, based on ITS sequences [52, 53]. The ITS sequence from *Z*. *mellis* type α, represented by strain NRRL Y-12628 (GenBank accession number AY046190), was 71.9% identical to *Z*. *mellis* ITS type β (strain NBRC 0485; GenBank accession number AB302839). *In silico* restriction analysis of ITS sequence types α and β resulted in two completely different genotypes, highly similar to the G-1 and G-2 genotypes, respectively (data not shown). This finding suggested that honey strains with genotype G-2 could harbor the ITS sequence type β. In addition to types α and β, another ITS genotype, named G-3, was observed in strains 9, 27, and 41, whereas strain 5CF was clearly different from any other honey strain in its unique restriction pattern (Table 1).

**Table 1:** ITS restriction fingerprinting and direct sequencing of ITS regions and 26S rDNA D1/D2 domains. Clusters G-1 to G-9 and singleton ITS profiles (-) are determined based on restriction digestion patterns of ITS amplicons with the endonucleases *Hae*III, *Hha*I and *Hinf*I. Restriction fragments lower than 70 bp are omitted from the analysis. Strains in bold are chosen as representatives of each cluster and/or unique ITS genotypes and are submitted to direct ITS and D1/D2 sequencing. Overlapping peaks in chromatograms due to ambiguous sites and/or indels are indicated as unreadable sequences. Closest-sequence matches are reported together with identity (%). Abbreviations: na, not applicable; nd, not determined; cp, copy.

Source	Strains	Amp (bp)	ITS PCR-RFLP			Group	Closest Blast hits (Accession number; identity %)	
			*Hae*III	*Hinf*I	*Hha*I		ITS	26S D1/D2
**Honey**								
	**2**,3, 12, 24, 56, 68, 76	900	570-200-80	380-280-180	300-220-190-100	**G-1**	*Z*. *mellis* type α (AY046190;100)	*Z*. *mellis* (U72164;99)
	**4**, 7, 23, 35, 40, 1CF, 6C	900	680–170	480-290-140	310-190-100	**G-2**	*Z*. *mellis* type β (AB302839;99)	*Z*. *mellis* (AB302835;100)
	**9**, 27, 41	900	390-250-170	480-290-140	300-190-100	**G-3**	nd	*Z*. *mellis* (U72164;99)
	**70**	900	680–170	480-290-140	380-320-130	**-**	no significant entries	*Z*. *mellis* (U72164;99)
	**5CF**	700	700	360-270-250-220-180	360-290-200-170-100	**-**	*D*. *hansenii* (EF192224;100)	*D*. *hansenii* (KF273863;100)
**Traditional Balsamic Vinegar**								
	**B8911**, B8932, B8933, B8941, B89221	700	390-210-90	360-250-180	290-200-170-100	**G-4**	*Z*. *rouxii* (AM943655;99)	*Z*. *rouxii* (AM943655; 100)
	**B8943**	650	420–150	450–280	300–290	**G-5**	*Z*. *bailii* (JX458100;100)	*Z*. *bailii* (AY046191; 99)
	**M21**, M22, M23, M25	700	510-480-390-250-210-170-90	380-360-260-250-220-180	360-290-200-170-100	**G-6**	unreadable sequence	*Z*. *sapae* (AJ966342;100)
***Shoyu* mash, *Shoyu moromi*, and *Miso***								
	**NBRC 0495**	700	480-440-220-170	360-250-220-110	320-290-200-170-100	**-**	unreadable sequence	unreadable sequence
	**NBRC 0505**, NBRC 0506, NBRC 0521, NBRC 0523	700	480-170-90	360-160-130	320-290-100	**G-7**	*Z*. *sapae* cp 2 (AM2794964;99)	unreadable sequence
	**NBRC 0525**	700	480-170-90	340-220-190	320-290-100	**-**	*Z*. *sapae* cp 2 (AM2794964;99)	unreadable sequence
	**NBRC 0845**, NBRC 0846	700	390-210-90	360-250-180	290-200-170-100	**G-4**	*Z*. *rouxii* (AM943655;99)	*Z*. *sapae* (AJ966342; 100)
	**NBRC 10652**, NBRC 10655	700	390-210-170-90	360-250-220-180	320-290-200-170-100	**G-8**	unreadable sequence	unreadable sequence
	**NBRC 10668**	700	390-170-90	360-250-220-180	320-290-100	**-**	CBS 4837 cp 2 (HE664090;99)	*Z*. *sapae* (AJ966342;100)
	**NBRC 10669**	700	480-390-210-90	360-280-250-220-180	290-200-170-100	**-**	unreadable sequence	unreadable sequence
	**NBRC 10670**	700	480-390-210-90	360-250-180	290-200-170-100	**-**	unreadable sequence	unreadable sequence
	**NBRC 10672**	700	480-390-250-210-170-90	360-250-220-150	290-200-170-100	**-**	unreadable sequence	unreadable sequence
**Reference strains**								
	*Z*. *bailii* CBS 680^T^	650	420–150	450–280	300–290	**G-5**	na	na
	*Z*. *mellis* CBS 736^T^	900	570-200-80	380-280-180	300-220-190-100	**G-1**	na	na
	*Z*. *rouxii* CBS 732^T^	700	390-210-90	360-250-180	290-200-170-100	**G-4**	na	na
	*Z*. *sapae* ABT301^T^, ABT601	700	510-480-390-250-210-170-90	380-360-260-250-220-180	360-290-200-170-100	**G-6**	unreadable sequence	na
	*Z*. *pseudorouxii* NCYC 3042	700	480-170-90	360-220-180	320-290-100	**-**	*Z*. *sapae* cp 2 (AM2794964;99)	*Z*. *sapae* (AJ966342;100)
	Allodiploid lineage ATCC 42981, CBS 4837, CBS 4838	700	480-390-250-210-170-90	380-360-250-230-180	360-290-200-170-100	**G-9**	unreadable sequence	unreadable sequence

Ten TBV strains divided into three groups, which we termed G-4, G-5 and G-6 based on ITS patterns (Table 1). The G-4 and G-5 patterns were identical to those of *Z*. *rouxii* and *Z*. *bailii* type strains, respectively, suggesting that homogenized *Z*. *rouxii* and *Z*. *bailii* ITS haplotypes were present in rDNA repeats of these groups. The G-6 pattern was identical to that of *Z*. *sapae* and consisted of extra bands arisen from divergent ITS haplotypes. This genotype slightly differed from the G-9 pattern found in strains belonging to mosaic lineage, such as ATCC 42981, CBS 4837, and CBS 4838 [31, 32].

ITS restriction analysis identified highly diverse genotypes among *Z*. *rouxii* NBRC strains. Two strains, namely NBRC 0845 and NBRC 0846, possessed the canonical *Z*. *rouxii* genotype G-4, whereas 6 strains displayed unique restriction patterns, and the remaining 6 strains showed the genotypes G-7 and G-8. These last genotypes did not match any pattern from reference strains used in this study or reported in published data (Table 1).

### Inter- and intra-individual variability of ITS haplotypes

Based on ITS restriction fingerprinting, one representative strain for each cluster and/or for each food source and 9 singleton strains were chosen for ITS sequencing (Fig 1B). Direct ITS sequencing was successful for 11 strains. BLAST searches for the ITS sequences of honey strains supported the high inter-individual variability in ITS1 and ITS2 spacers within the clade *Z*. *mellis*/*Z*. *siamensis*. In particular, strain 2 (genotype G-1) exhibited ITS type α (100% identity with CBS 736^T^), while strain 4 showed ITS type β (99% identity with NBRC 0485). Singleton strain 70 displayed a unique ITS sequence divergent from any other ITS sequence deposited in GenBank (86% and 85% similarities compared to *Z*. *mellis* β-type NBRC 0485 and *Z*. *siamensis* JCM 16825, respectively). The honey strain 5CF was 100% similar to *Debaryomyces hansenii*, a species frequently isolated from raw honey and bee-related environments [54].

Direct ITS sequencing was also successful for strains B8943 (100% identity with *Z*. *bailii*) and B8911 (100% identity with *Z*. *rouxii*), as well as for some NBRC strains (Table 1). In particular, strain NBRC 0845 had an ITS sequence 99% identical with *Z*. *rouxii*, in agreement with the RFLP pattern. A YeastIP search showed that ITS sequences from strains NBRC 0525 and NBRC 0505 match ITS haplotype 2 from *Z*. *sapae* ABT301^T^ (99% identity) [36, 41], whereas strain NBRC 10668 possesses a unique ITS sequence 99% identical to ITS variant 2 previously cloned in mosaic strain CBS 4837 [31, 32].

Out of 17 strains analyzed, six strains failed in direct ITS sequencing (M21, NBRC 0495, NBRC 10652, NBRC 10669, NBRC 10670 and NBRC 10672). According to multi-band patterns scored by ITS-RFLP, chromatographs showed overlapping peaks corresponding to ambiguous nucleotides and/or indels, indicating the presence of different ITS copy variants in an individual genome. To verify intra-individual polymorphisms, the PCR-amplified ITS fragments were cloned from gDNAs of single-colony cultures. A summary of these ITS cloning experiments is reported in S3 Table. Intra-individual polymorphisms were positively identified only when each intra-genomic ITS version was present in at least two clones per strain. Pairwise comparisons of full-length intra-individual ITS regions showed that any of these strains possesses two highly divergent ITS types (designated as copies 1 and 2, respectively), which shared 95.73 to 91.00% of sequence identity (Table 2). When ITS1 and ITS2 segments were analyzed separately, we scored three strains (NBRC 0495, NBRC 10652, and NBRC 10672) which retain polymorphisms only in ITS2, and one strain (NBRC 10670) which have 46 substitutions and 5 indel only in ITS1 segment (Table 2). Intra-genomic ITS variant of strain M21 exhibited the lowest level of sequence identity, with substitutions and indels both in ITS1 and ITS2 segments. The rRNA secondary structures of cloned ITS1 and ITS2 transcripts overlap those detected in *Z*. *rouxii*, *Z*. *sapae*, and *Z*. *mellis* and exclude that the intra-genomic variants are pseudogenes (data not shown).

**Table 2:** Pairwise comparisons between intra-genomic ITS1 and ITS2 variants cloned from each strain. Numbers of transition (s_i_) and transversion (s_v_), as well as the number of indels are computed in pairwise comparisons between copies 1 and 2 of ITS1 and ITS2 segments with MEGA6. The total number of nucleotides in indels is reported in brackets. Identity (%) indicates percentage of identical nucleotides between copies 1 and 2 of full-length ITS region or ITS1/ITS2 segments scored within each strain. Abbreviation: cp, copy.

Strains	N°cp	cp1 *vs* cp2 identity (%)			ITS1				ITS2	
			S_i_	S_v_	Indel (nt)	Identity (%)	S_i_	S_v_	Indel (nt)	Identity (%)
NBRC 0495	2	94.21	0	0	0	100	17	21	6(66)	85.3
NBRC 10652	2	94.98	0	0	0	100	18	20	5(42)	86.73
NBRC 10669	2	94.48	20	24	6(35)	81.37	3	1	2(14)	98.20
NBRC 10670	2	95.73	21	25	5(34)	80.79	0	0	1(1)	100
NBRC 10672	2	95.15	0	0	0	100	14	20	5(40)	87.32
M21	2	91.00	19	19	7(32)	83.08	14	10	7(57)	89.04

A YeastIP search reveals that the closest relatives of full-length intra-genomic ITS variants are from *Z*. *rouxii*, *Z*. *sapae*, mosaic lineage of the *Z*. *rouxii* complex and *Z*. *mellis* (S3 Table). Different combinations of ITS haplotypes were scored within individual genomes. In strains NBRC 10652 and NBRC 10672, ITS haplotypes 1 and 2 were 99% identical to *Z*. *rouxii* and CBS 4837 haplotype 2 [31, 32], respectively (S3 Table). Strain NBRC 10670 displayed one haplotype 100% identical to *Z*. *rouxii* and another 99% identical to ITS haplotype 3 from mosaic strain CBS 4838 [31]. ITS haplotypes 1 and 2 of strain NBRC 0495 were 99% identical to CBS 4838 haplotype 3 and *Z*. *sapae* haplotype 2, respectively. In strain NBRC 10669, ITS variants 1 and 2 were 99% identical to haplotypes 2 of mosaic strain CBS 4837 and *Z*. *sapae* ABT301^T^, respectively. ITS variants 1 and 2 from strain M21 (representing ITS genotype G-8 identical to *Z*. *sapae*) were 98% identical to *Z*. *sapae* ITS copies 1 and 2, respectively. Because out of 20 screened clones from strain M21, none showed a *Hae*III restriction pattern diagnostic of ABT301^T^ ITS copy 3, we concluded that strain M21 lacks ITS variant 3 previously found in *Z*. *sapae* [37] (S3 Table).

To estimate intra- and inter-strain ITS variation, we performed a multiple sequence alignment of 29 ITS1 and ITS2 sequences, collected here and in previous studies [32–34] (S1 and S2 Files). The majority of polymorphic sites were distributed on both the spacers, with polymorphic sites in ITS2 generally outnumbering those in ITS1. In particular, the length of ITS1 ranged from 216 to 231 bp (237 as alignment length), with 43 variable characters, 39 parsimony-informative characters, and 4 singletons. Gaps or missing data represented 24 sites, and the GC content ranged from 16.87–18.65% (17.65% average). The length of ITS2 ranged from 224 to 273 bp (289 as alignment length), with 125 variable characters, 119 parsimony-informative characters, and 6 singletons. Gaps or missing data represented 75 sites, and the GC content ranged from 18.81–20.93% (19.71% average). The 5.8S rRNA gene sequences were 156 bp long and showed a single nucleotide transition A/G in position 131 bp (numbering refers to *Z*. *rouxii* 5.8S rDNA) across the entire dataset.

### Phylogenetic and network analysis based on ITS regions

**Fig 2:**
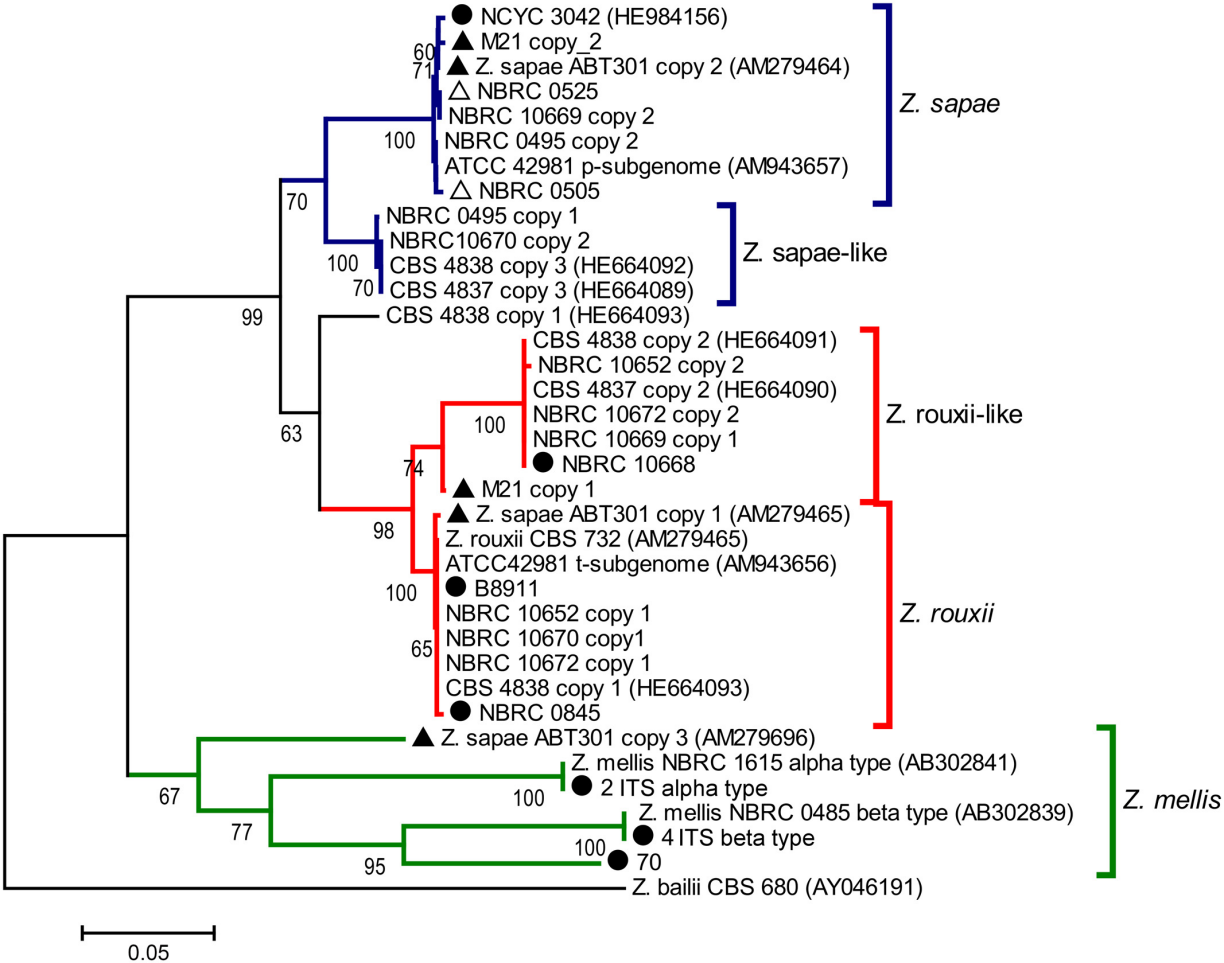
Phylogenetic relationships within the *Z*. *rouxii* complex inferred from ITS sequences. This phylogeny was inferred through the Neighbor-Joining method using the ITS sequence of *Z*. *bailii* CBS 680^T^ (GenBank accession number AY046191) as outgroup. The percentage values of replicate trees in which the associated taxa clustered together in the bootstrap test (10,000 replicates) are shown next to the branches (only values higher than 60% are reported). The evolutionary distances were computed using the Tamura-Nei method [47]. The tree is drawn to scale, with branch lengths in the same units as those of the evolutionary distances used to infer the phylogenetic tree. All positions containing gaps and missing data were eliminated. *Z*. *rouxii* and *Z*. *rouxii*-like clades are colored in red, *Z*. *mellis* clade in green, and *Z*. *sapae* and *Z*. *sapae*-like clades in blue. Black triangles represent strains with homogeneous 26S rDNA D1/D2 domains and heterogeneous ITS regions. Black circles indicate strains without any rDNA heterogeneity. White triangles indicate strains with heterogeneous 26S rDNA D1/D2 domains and homogeneous ITS rDNA regions.

To investigate the genealogical relationships among the ITS haplotypes, a dataset was generated that included 6 GenBank sequences of related *Zygosaccharomyces* species and 29 sequences of *Z*. *rouxii* complex yeasts obtained both by cloning and direct sequencing. The dataset was used to construct a NJ phylogenetic tree (Fig 2). The tree topology was congruent with the delineation of three major branches, referred to as *Z*. *sapae*, *Z*. *rouxii* and *Z*. *mellis*. The *Z*. *rouxii* and *Z*. *sapae* branches included two minor branches, termed *Z*. *rouxii*-like (74% bootstrap support) and *Z*. *sapae*-like clusters (100% bootstrap support). The only sequence that does not clearly belong to one group or the other is CBS 4838 copy 1. The *Z*. *mellis* branch is further divided into different sub-branches, including α, β ITS types and additional divergent ITS variant cloned from *Z*. *sapae*. With the exception of CBS 4838 ITS copy 1, intra-genomic ITS variants cloned from any single strain did not form homogeneous clusters, but were instead split across the *Z*. *sapae*, *Z*. *rouxii* and *Z*. *mellis* branches.

**Fig 3:**
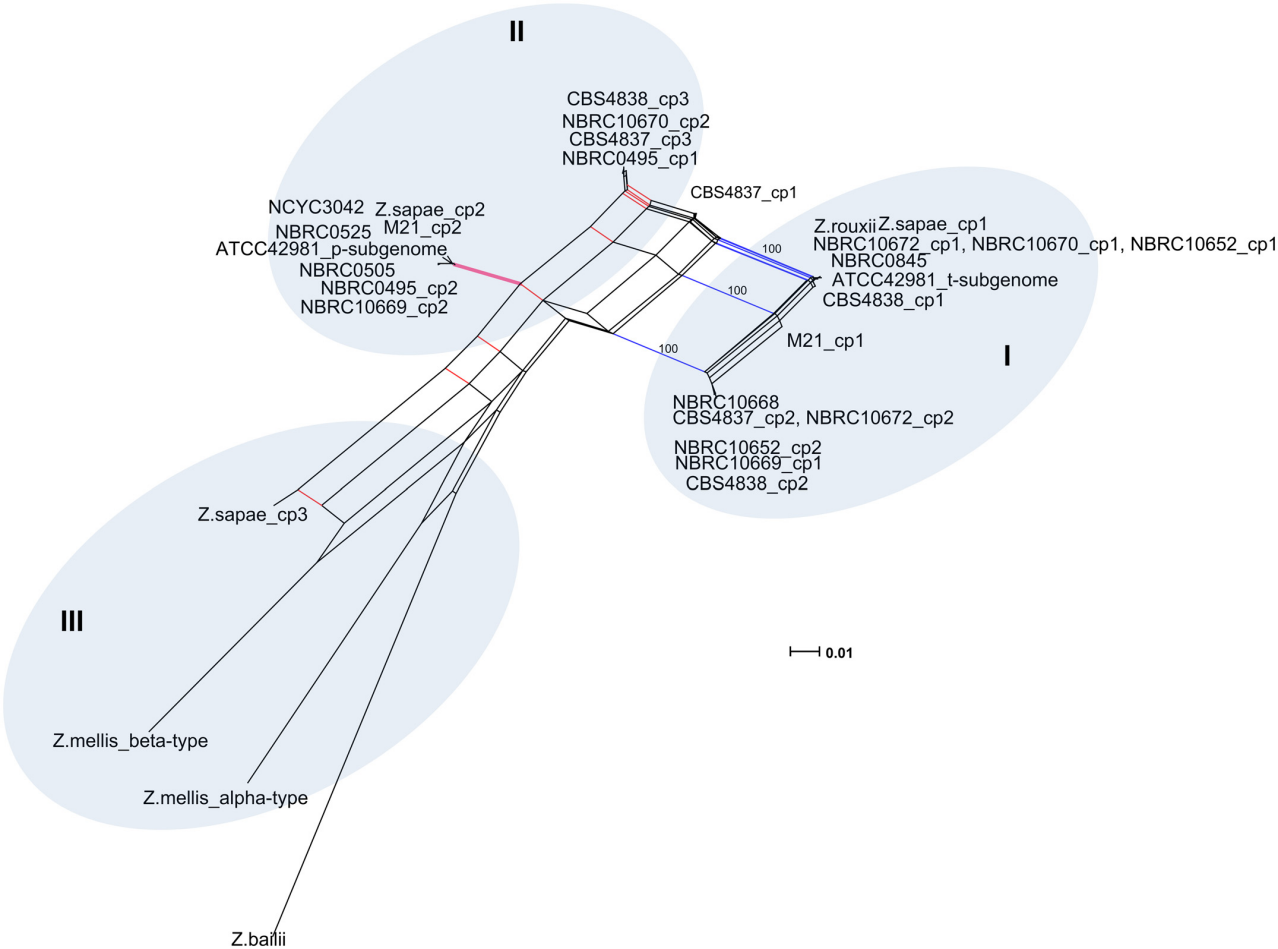
Neighbor-net network of ITS sequences. Both intra- and inter-strain variants are considered. For display purposes, bootstrap scores are not shown. The scale bar represents the split support for the edges. Blue and red edges mark splits separating the three major clusters. Clusters described in the text are denoted by Roman numerals.

Overall these evidences suggested that rDNA arrays did not evolve in a tree-like way, but rather in a reticulate way that cannot be represented by a bifurcating tree ([51] and references therein). Reticulation can be further confirmed if intra-genomic ITS copies present in any individual are less similar to one another than to a sequence present in another related species [55]. Accordingly, we found that the evolutionary divergence between intra-individual haplotypes was greater than the value found between each ITS variant and the most similar ITS sequence in another species, indicating likely the generation of additional variants via reticulation (S4 Table). Consequently, a sequence dataset including intra- and inter-genomic ITS variants within the *Z*. *rouxii* complex, was used as input for the SplitsTree program, and a reticulate network tree was drawn using the Neighbor-net algorithm. The unrooted network generated by Neighbor-net showed a distinctly non-treelike topology (Fig 3). The *Z*. *bailii* sequence used as outgroup in the NJ analysis was also included in the Neighbor-net analysis, as its inclusion had little effect on the overall structure of the network topology (the Neighbor-nets are unrooted networks) (data not shown). When examining the output from the SplitsTree analysis, more splits were observed. The networked relationships among the sequences showed box-like structures instead of bifurcations, confirming that reticulation has taken place during the evolution of the *Z*. *rouxii* complex. The Phi test also showed evidence for recombination (*p* = 0.0), and the average Delta score (0.2416) and the average Q-residual (0.09826) supported the network-like layout of the dataset. Even though the network was highly netted, distinct clusters could be discerned. Edges marked in blue and red delineated three clusters (Fig 3). Cluster I contained all the *Z*. *rouxii*-related sequences, including *Z*. *sapae* copy 1 and the intra-genomic ITS copy 1 variants cloned from heterogeneous strains. Cluster II consisted of ITS haplotypes 2 and 3, whereas cluster III grouped the *Z*. *mellis*-related sequences, including the divergent ABT301^T^ ITS copy 3.

### Inter- and intra-individual variability of 26S rDNA D1/D2 domains

We sequenced the 26S rDNA D1/D2 domains of 17 strains (8 strains representative of ITS genotypes G-1 to G-8 and 9 singleton strains). Five honey strains (namely 2, 4, 9, 41, and 70) displayed D1/D2 sequences 99–100% identical to that of *Z*. *mellis* without any evidence of intra-genomic heterogeneity in this portion of rDNA units (Table 1). These data indicate that divergent inter-individual ITS haplotypes co-exist with highly conserved D1/D2 domains in the rDNA arrays of *Z*. *mellis* strains. Based on both ITS and D1/D2 sequences, strains B8911 and B8943 were assigned to the species *Z*. *rouxii* and *Z*. *bailii*, respectively. Although strains M21, NBRC 10668, and NBRC 0845 differed from each other and from *Z*. *sapae* ABT301^T^ in ITS regions, they possessed the same *Z*. *sapae* D1/D2 sequence (Table 1).

Direct sequencing of D1/D2 domains resulted in unresolved nucleotides for 7 strains purchased from NBRC. To exclude the possibility of contaminated cultures, the sequencing was carried out using template DNA extracted from cultures after two rounds of streaking. To examine the reasons why ambiguous positions occur in these chromatograms, we cloned PCR-amplified D1/D2 fragments. Based on polymorphic sites among the of *Z*. *rouxii* (r), *Z*. *sapae* (s), and *Z*. *mellis* (m) D1/D2 sequences, the endonuclease *Ava*I was chosen as the diagnostic restriction enzyme to differentiate the D1/D2 substitutions among these species (S5 Table). Based on *Ava*I-RFLPs, two different D1/D2 clone fragments were identified in 5 NBRC strains, whereas three and four D1/D2 variants were detected in strains NBRC 0505 and NBRC 10669, respectively (S5 Table). In particular, Suezawa et al. [52] did not detect any intra-genomic polymorphisms at the D1/D2 segments from strains NBRC 0525 and NBRC 0505. To ensure that nucleotide differences did not arise from PCR and cloning errors, additive polymorphisms were positively identified only when each of the versions was present in at least two clones per strain.

Pairwise comparisons were performed between intra-genomic D1/D2 variants derived from any single strain. As reported in Table 3, the differences between intra-genomic D1/D2 variants exceeded 1%, the value generally considered as the limit of variability among conspecific yeast strains [56, 57]. In particular, strain NBRC 0495 showed the highest number of substitutions between D1/D2 haplotypes and the lowest level of intra-genomic identity (92.03%), whereas, in strains NBRC 0525, NBRC 10652, NBRC 10670, and NBRC 10672, sequence identity between intra-genomic D1/D2 haplotypes ranged from 96.50 to 97.51%. In strain NBRC 0505, the D1/D2 variant m was 95.45 and 94.59% identical to variants s and r, respectively. In strain NBRC 10669, four D1/D2 types were cloned, namely m, r, r* and s. D1/D2 variant r* differed from variant r only by 4 SNPs (Table 3). In particular, one G-to-A transition removed an *Ava*I restriction site in variant r* compared to variant r.

**Table 3:** Pairwise comparison of D1/D2 sequences cloned from strains with intra-genomically variable D1/D2 sequences. Intra-genomic D1/D2 variants cloned from individual strains are indicated as D1/D2 copies. The number of transitional (s_i_) and transversional (s_v_) mutations, as well as number of indels and the involved nucleotides, were computed with MEGA6. The number of nucleotides in pairwise-aligned sequences is indicated as nt (tot), whereas the number of nucleotides in indels (N° nt) is reported in brackets. Identity (%) indicates the percentage of identical nucleotides between D1/D2 variants within individual genomes.

Strain	D1/D2 copies		nt (tot)	Polymorphic sites				Identity (%)
	N°	Comparison		tot	s_i_	s_v_	Indel (N° nt)	
NBRC 0495	2	copy m *vs* copy s	581	47	24	22	3(6)	92.03
NBRC 0505								
	3	copy m *vs* copy s	578	28	21	7	3(6)	96.15
		copy m *vs* copy r	578	31	21	10	3(5)	94.59
		copy s *vs* copy r	573	14	9	5	1(2)	97.55
NBRC 0525	2	copy r *vs* copy s	572	7	10	5	1(1)	97.02
NBRC 10669								
	4	copy m *vs* copy s	578	21	15	6	3(6)	95.45
		copy m *vs* copy r*	578	32	22	10	3(4)	94.43
		copy m *vs* copy r	578	30	21	9	3(4)	94.59
		copy s *vs* copy r*	574	15	10	5	2(2)	97.38
		copy s *vs* copy r	573	15	11	4	1(1)	97.38
		copy r *vs* copy r*	574	4	3	1	1(1)	99.38
NBRC 10652	2	copy s *vs* copy r	573	15	10	5	1(1)	97.38
NBRC 10670	2	copy s *vs* copy r	573	20	12	8	1(1)	96.50
NBRC 10672	2	copy s *vs* copy r	563	14	9	5	1(1)	97.51

BLAST searches in NCBI and YeastP databases showed that the closest relatives to the cloned intra-genomic D1/D2 variants are from *Z*. *rouxii*, *Z*. *sapae*, and *Z*. *mellis* species (S5 Table). The majority of strains exhibit two intra-genomic D1/D2 haplotypes 99% identical to *Z*. *rouxii* and *Z*. *sapae*, respectively. As exception, strain NBRC 0495 displayed one *Z*. *sapae* D1/D2 copy s and another D1/D2 copy m that did not match any entry in public databases (92% identity to *Z*. *mellis*). In heterogenous strains NBRC 0505 and NBRC 10669, the most identical database entries to D1/D2 variants r, s, and m were *Z*. *rouxii* (99%), *Z*. *sapae* (99%) and *Z*. *mellis* (99%), respectively. The 2D structures of D1 and D2 domains conserve the same hairpin stem topologies of the *Z*. *rouxii*, *Z*. *sapae*, *Z*. *mellis* counterparts, excluding that cloned intra-individual variants are pseudogenes and/or PCR chimeras (data not shown).

Multiple sequence alignment of all cloned D1/D2 sequences identified 55 variable positions, in two short regions of domains D1 and D2, namely VR1 and VR2 (S3 and S4 Files). VR1 resides in the core of the large rRNA subunit, while VR2 corresponds to a short stretch of an expansion segment of the *S*. *cerevisiae* D2 domain which is missing in the *E*. *coli* LSU rRNA molecule [58]. While the core regions of the LSU rRNAs are structurally conserved across all domains of life, the expansion segments evolve more rapidly [59], presumably due to reduced functional constraints. Accordingly, 11 variable nucleotides were detected in VR1 (whereof 9 were parsimony-informative and 2 were singletons) and 44 in VR2 (whereof 19 were parsimony-informative and 25 were singletons). Usually, transitions are generated at higher frequency than transversions, and two nucleotides alternate. There were only one site in VR1 and four sites in VR2 where more than two different nucleotides occurred when all cloned sequences were compared.

### Phylogenetic and network analysis based on 26S rDNA D1/D2 domain

**Fig 4:**
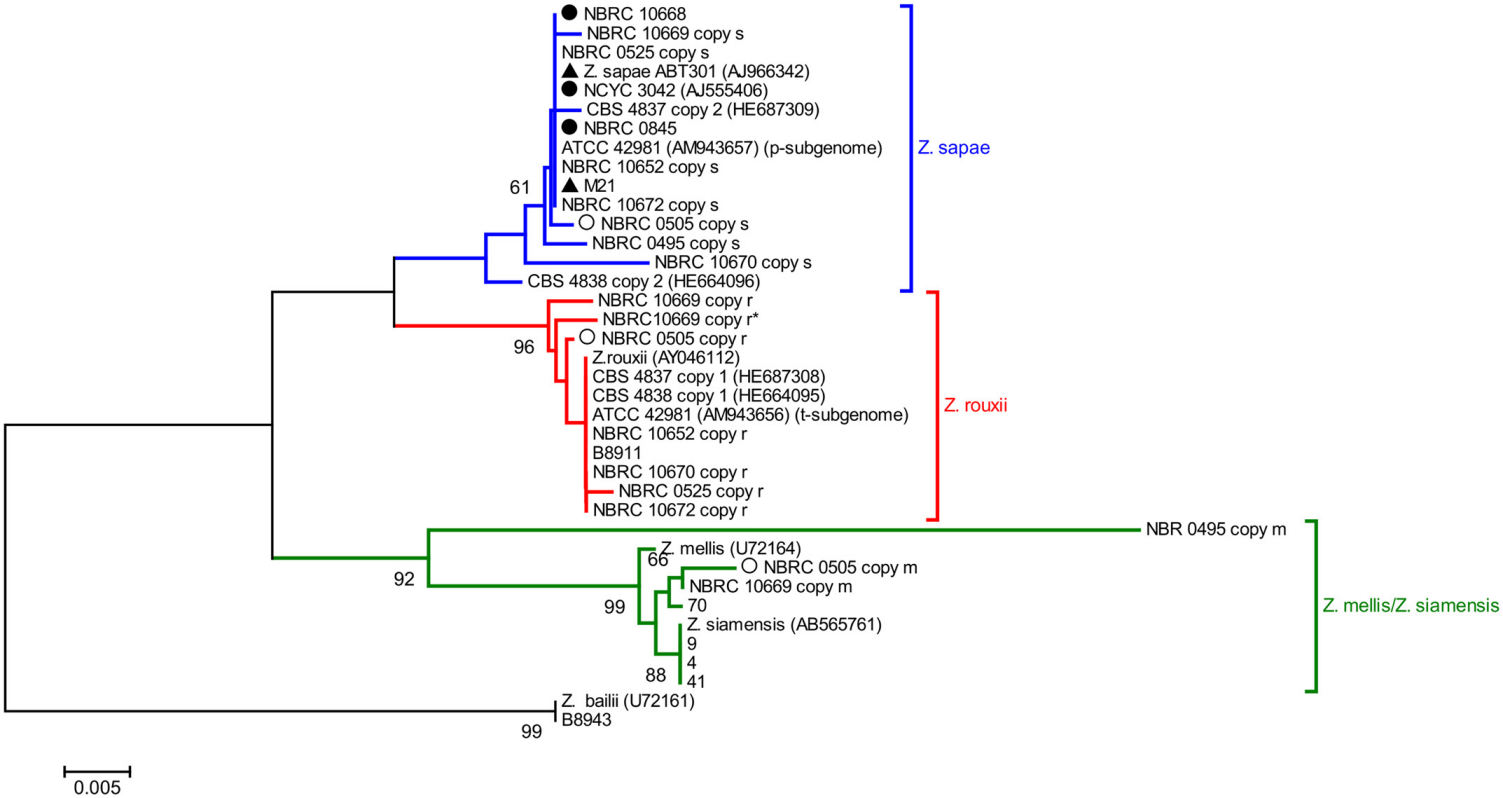
Phylogenetic relationships within the *Z*. *rouxii* complex based on 26S rDNA D1/D2 sequences. This phylogeny was inferred using the Neighbor-Joining method using D1/D2 sequence of *Z*. *bailii* CBS 680^T^ (GenBank accession number U72161) as outgroup. The percentages of replicate trees in which the associated taxa clustered together in the bootstrap test (10,000 replicates) are shown next to the branches (only values higher than 60% are reported). The tree is drawn to scale, with branch lengths in the same units as those of the evolutionary distances used to infer the phylogenetic tree. The evolutionary distances were computed using the Tajima-Nei method [48]. All positions containing gaps and missing data were eliminated. *Z*. *rouxii*, *Z*. *sapae*, and *Z*. *mellis* clades are colored in red, blue, and green, respectively. Black triangles represent strains with homogeneous 26S rDNA D1/D2 domains and heterogeneous ITS regions. Black circles indicate strains without rDNA heterogeneity. White triangles indicate strains with heterogeneous 26S rDNA D1/D2 domains and homogeneous ITS rDNA regions.

The phylogenetic tree was built on a dataset of 39 D1/D2 sequences from 26 *Zygosaccharomyces* strains, using *Z*. *bailii* as outgroup (Fig 4). The NJ-tree grouped the sequences into three lineages with support > 60%, hereafter referred to as *Z*. *sapae*, *Z*. *rouxii*, and *Z*. *mellis*/*Z*. *siamensis*. The lineage *Z*. *sapae* comprised 5 D1/D2 haplotypes obtained by direct sequencing (M21, NBRC 10668, NBRC 0845, NCYC 3042, and *Z*. *sapae* ABT301^T^ strains) and 9 D1/D2 haplotypes from heterogeneous strains (ATCC 42981, CBS 4837, CBS 4838, NBRC 0495, NBRC 0505, NBRC 0525, NBRC 10652, NBRC10669, and NBRC 10670). This cluster is not well separated from two additional clusters containing *Z*. *mellis*/*Z*. *siamensis* and *Z*. *rouxii* D1/D2 rDNA haplotypes, respectively. In the *Z*. *rouxii* lineage, 9 out of 11 sequences were cloned from heterogeneous strains, whereas the lineage *Z*. *mellis*/*Z*. *siamensis* included 4 honey strains that clustered together regardless of the inter-individual ITS diversity. D1/D2 haplotype m from strain NBRC 0495 clustered differently from other members of the *Z*. *mellis*/*Z*. *siamensis* clade. Significantly, intra-genomic D1/D2 variants from single individuals assorted in different clusters and displayed values of within-strain divergence higher than the values of evolutionary divergence measured by comparing each D1/D2 variant with the closest strain/species (S6 Table). Overall these evidences suggest that reticulation took place in evolutionary history of the D1/D2 segment.

**Fig 5:**
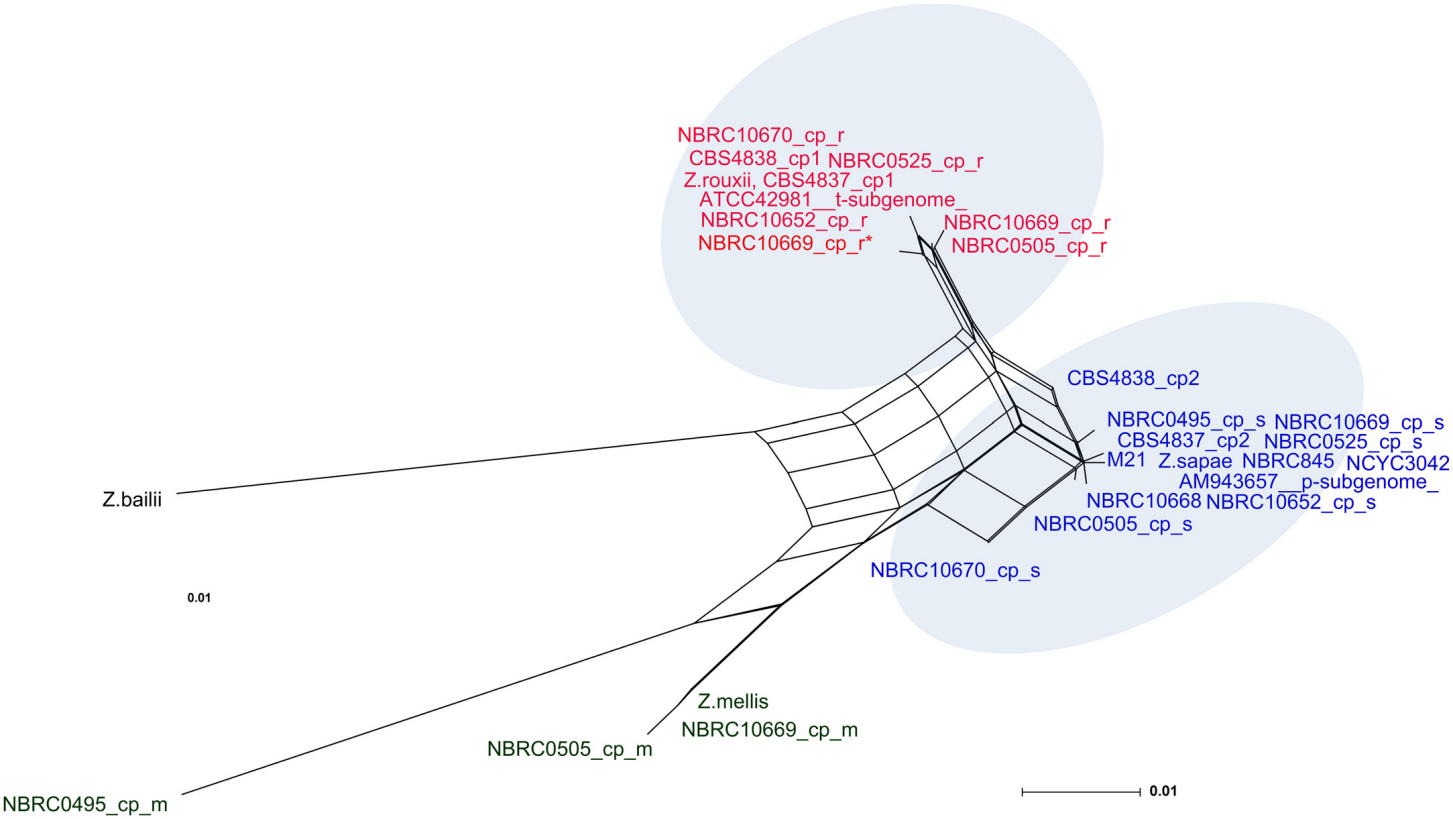
Neighbor-net network of D1/D2 sequences. A dataset of 30 intra-genomic and inter-strain variants is considered. The scale bar represents the split support for the edges. For display purposes, bootstrap scores are not shown. Strains belonging to *Z*. *sapae* and *Z*. *rouxii* clusters are denoted by red and blue labels, respectively.

As reported above, network approaches provide a better indication of reticulate relationships in a given dataset than standard phylogenetic trees. Therefore, a subset of 30 D1/D2 sequences cloned here and in our previous works [31, 32] was selected to generate a reticulate network tree of the *Z*. *rouxii* complex (Fig 5). The high number and the directions of network edges, as well as the Delta score and the Q-residual (Delta score = 0.2175; Q-residual = 0.02131) clearly demonstrate that the dataset is largely network-like. Although several alternative phylogenetic histories emerge from the Neighbor-net tree (Fig 5), we observed that s and r variants are concentrated on the right side of the diagram, while m variants are mostly present on the left side, which is the part of the network with the longest branches. In particular, the same cloned sequences that formed the branches *Z*. *sapae*, *Z*. *rouxii*, and *Z*. *mellis*/*Z*. *siamensis* in the phylogenetic NJ-tree (Fig 4) also grouped together in the phylogenetic network (Fig 5), but their lineages were interconnected through network edges. The internal nodes in this network represent ancestral species, and nodes with more than two parents correspond to ‘reticulate’ events, such as recombination. Recombination was also supported by Phi test (*p* = 2.615 x 10^−4^).

## Discussion

### Cataloguing ribosomal heterogeneity in the *Z*. *rouxii* species complex

**Fig 6:**
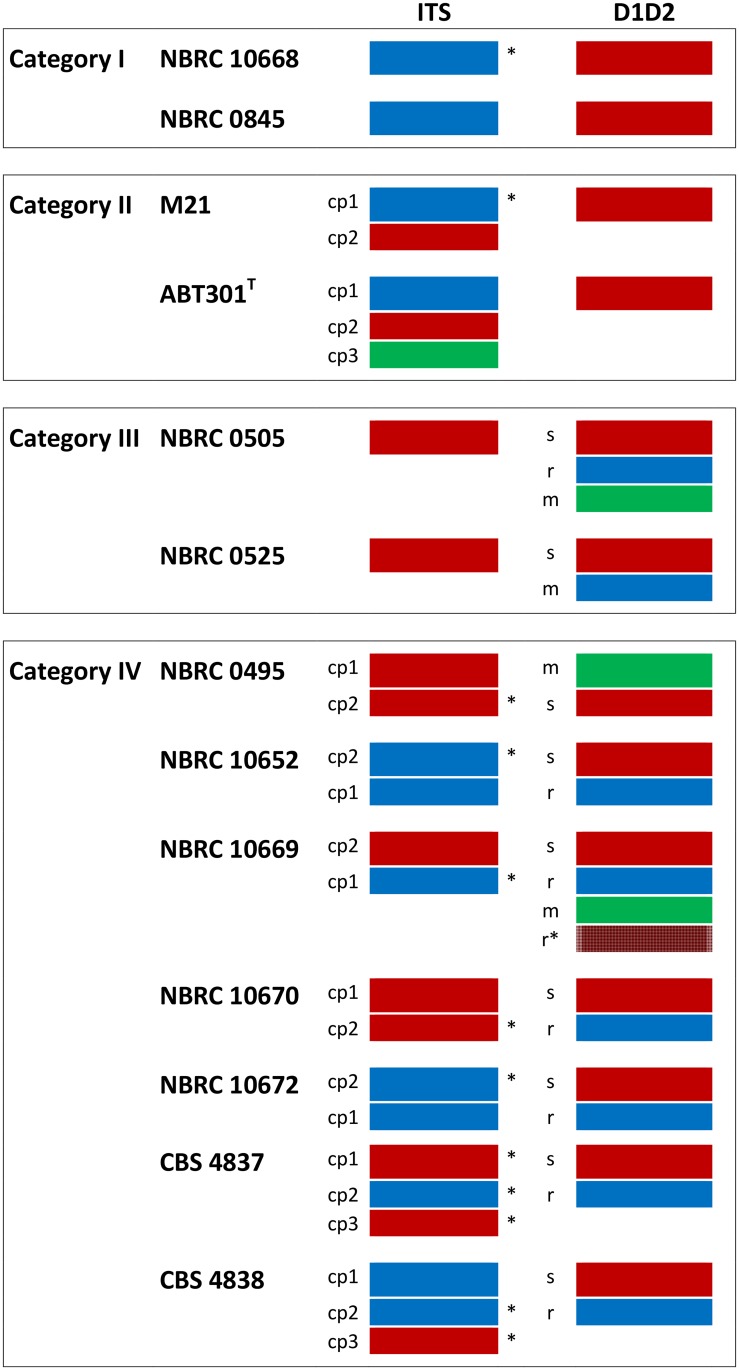
Patterns of ribosomal homogenization detected in *Z*. *rouxii* complex strains. Four evolutionary outcomes (or categories) result from homogenization of divergent intra-genomic ribosomal variants. Red, blue and green rectangles mark *Z*. *sapae*, *Z*. *rouxii* and *Z*. *mellis* ribosomal sequences, respectively; asterisks mark ITS sequences partially divergent from *Z*. *sapae* and *Z*. *rouxii* (minor clusters *Z*. *sapae*-like and *Z*. *rouxii*-like, according to Fig 2). r, s, and m indicate *Z*. *rouxii*, *Z*. *sapae*, and *Z*. *mellis* D1/D2 copy variants, respectively. Abbreviation: cp, copy.

In this study, we collected a non-redundant, large set of strains belonging to the *Z*. *rouxii* species complex and we demonstrated that intra-genomic rDNA variation is unusually widespread within this clade (15 strains out of 43 analyzed). By sequencing two clones for any rDNA variant, we adopted a conservative approach to ensure that the uncovered intra-individual polymorphisms were not due to technical artefacts, such as PCR chimeras or sequencing errors, even though the observed variations are underestimate of the true level of variations. The tree topologies of ITS and D1/D2 sequences delineate three major clusters, *Z*. *rouxii*, *Z*. *sapae* and *Z*. *mellis*, which share a common ancestor, and evolved in a reticulate–like fashion. Hybridization is described as a major cause accounting for reticulation in eukaryote genome evolution and for rDNA heterogeneity in fungi. Two additive parental rDNA repeats are expected to be retained in yeast hybrid genome. In *Z*. *rouxii* species complex, ribosomal heterogeneity displays puzzling patterns of complexity which partially overlap with the hybridization hypothesis. Based on the distribution of polymorphic sites and the high variability of the intra-individual variants, we inferred that homogenization differently recombines intra-genomic variants in diverse individuals, leading to four evolutionary outcomes (or categories) (Fig 6).

In category I (strains NBRC 0845 and NBRC 10668), no traces of rDNA heterogeneity were found. The rDNA array consists of tandem repeats of a homogenized chimeric DNA unit where *Z*. *rouxii* ITS sequences are intermixed with *Z*. *sapae* D1/D2 sequences. This chimeric rDNA array organization resembles that previously described for aneuploid strain OUT 7136, a member of allodiploid lineage within the *Z*. *rouxii* complex [31]. Although the current data set is too small to infer evolutionary mechanisms, we speculate that inter-array sequence homogenization successfully acted across phylogenetically divergent repeats, leading to a recombinant rDNA type.

In categories II (strain M21 and *Z*. *sapae*), intra-genomic polymorphisms are not uniformly distributed across the repeat but are located in regions under looser functional constraints, such as ITS regions (Fig 6). As result, polymorphic ITS sequences are intermixed with homogenized *Z*. *sapae* D1/D2 sequences, and the degree of intra-genomic ITS variability is high as or higher than the overall interspecific divergence. This biased homogenization of ITS regions has been frequently documented in fungi [17, 23–26, 60] and may reflect rounds of incomplete homogenization with strong selection for functional coding regions and partially relaxed selection on ITS.

In category III (NBRC 0505 and NBRC 0525), biased homogenisation of D1/D2 domains is coupled to homogenized ITS regions. The majority of polymorphic sites are located in segments VR1 and VR2, that fold back to form hairpin loops in the predicted 2D structure of the LSU rRNA. Intra-genomic variations also occur at the same positions in *Clavispora lusitaniae* [61] and *Metschnikowia* spp. [62]. In contrast, *S*. *cerevisiae* displays a few intra-genomic polymorphisms in D1/D2 domains, even when significant rDNA polymorphisms are detected in other ribosomal regions [63]. The lack of homogenization at the 26S rRNA gene indicates that homogenization of ancestral polymorphisms is more efficient in ITS than in D1/D2 regions. This distribution of intra-genomic polymorphisms is unusual and disagrees with the general observation that coding regions (e.g., the 18S and 26S rRNA genes) are homogenized much more rapidly than non-coding regions [63].

In category IV polymorphic sites are retained in both segments of the 35S cistron (Fig 6). NBRC 10669 is a a-mating type strain which possesses multiple divergent rDNA arrays where two ITS haplotypes have intermixed with four different D1/D2 variants. This finding indicates that homogenizing forces are considerably reduced at the rDNA loci of this strain, maintaining multiple rDNA copies within a single individual. Strains NBRC 10670 and NBRC 10672 exhibit an equal number of *Z*. *sapae* and *Z*. *rouxii* ITS and D1/D2 haplotypes, suggesting that they are hybrids which maintained biparental rDNA arrays after hybridization. However, as both strains have been described to possess a single *MAT*α locus (S1 Table), they are likely to be heterothallic and haploid. The presence of intra-genomic rDNA variants in putative haploid genomes suggests that divergent haplotypes did not arise from hybridization events and that the hybridization is not the only event leading to the observed ribosomal heterogeneity in *Z*. *rouxii* complex.

In the NBRC database, strain NBRC 10652 is described as segregant of a hybrid between heterothallic parental strains CBS 4837 and CBS 4838. The rDNA arrays of these parental strains are polymorphic both in ITS and D1/D2 segments [31], suggesting that intra-individual rDNA variation in NBRC 10652 does not arise from hybridization. However, heterogeneity alone cannot rule out the possibility that the parental strains themselves were hybrids. While parental strains CBS 4837 and CBS 4838 display three ITS variants and two divergent D1/D2 domains, the hybrid segregant NBRC 10652 has lost ITS variant 3. Because heterothallic *Zygosaccharomyces* strains undergo mating before meiosis [31], ITS variant 3 should be lost after zygote formation during a meiotic event. We argue that meiotic recombination and/or independent segregation of the chromosomes harboring divergent rDNA arrays contributed to the asymmetric loss of parental ITS variant 3 in strain NBRC 10652.

### Hypotheses about the origin of rDNA heterogeneity

In plants and other fungi, ribosomal heterogeneity has been attributed to the following causes: (i) homoeology, i.e. the persistence of two or more independently inherited arrays of 35S rDNA as found for allodiploids and allopolyploids with more than one NOR; (ii) incomplete concerted evolution among the multiple copies of 35S rDNA located within the same NOR; (iii) pseudogenes, i.e. the occurrence of non-functional ribosomal genes and/or non-functional copies of ITS spacer regions that do not allow for the proper processing of the primary transcript, and (iv) gene paralogy in a strict sense, *i*.*e*. the existence of several rDNA loci coding for functionally differing rRNAs. Whereas rDNA homoeology, incomplete concerted evolution, and pseudogenes have been documented for the 35S rDNA cistron, paralogs have been observed only for the 5S rRNA genes in filamentous fungi [17]. Divergent rDNA sequences might be pseudogenes, as described in dinoflagellates [64], but this hypothesis can be ruled out in the *Z*. *rouxii* complex, because polymorphisms, even elevated ones, do not change the ribosomal secondary structures (data not shown).

**Fig 7:**
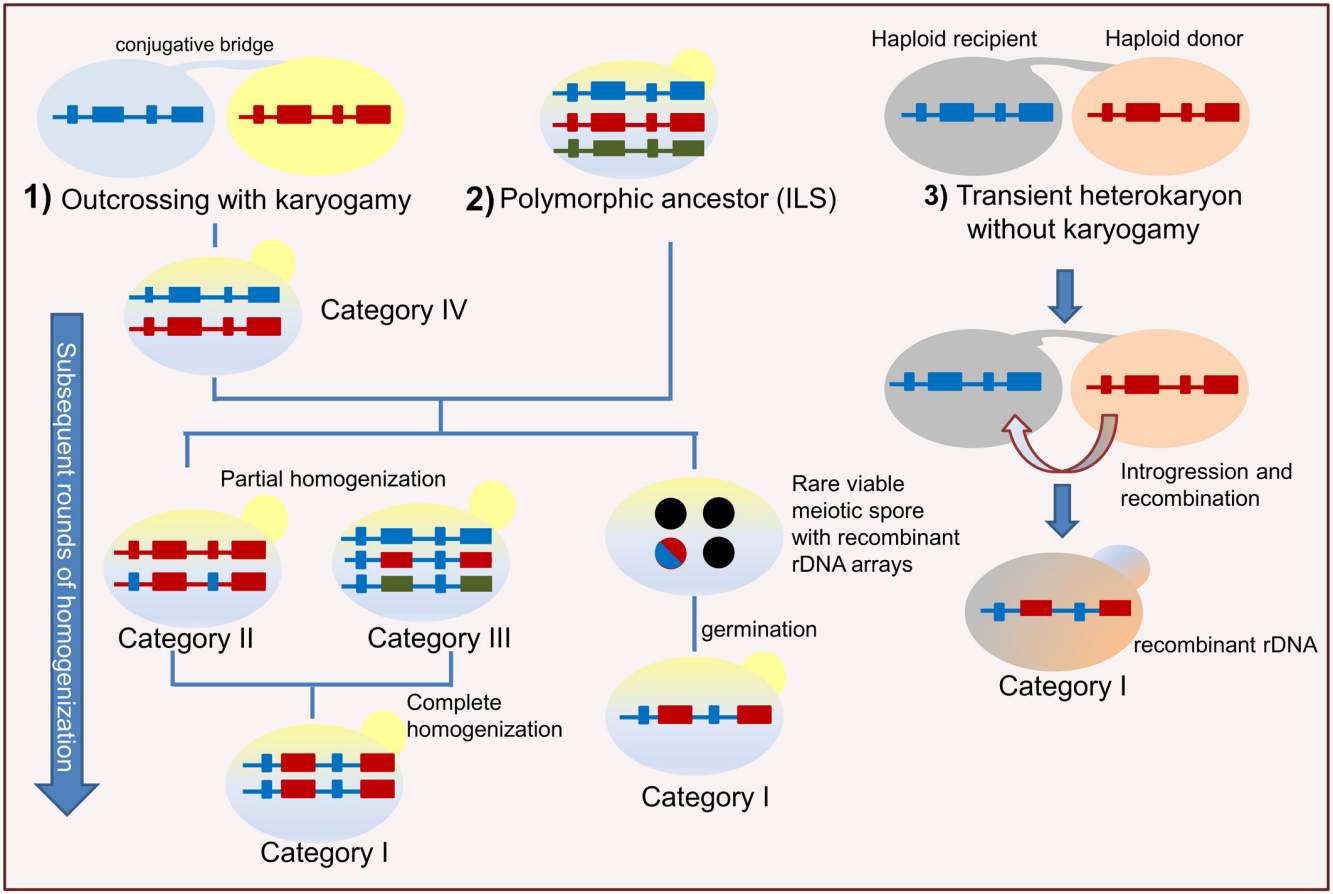
Model for the rDNA evolution in the *Zygosaccharomyces rouxii* complex. A schematic course of the evolution in rDNA arrays is shown. Small rectangles represent ITS regions, whereas the big ones 26S rRNA genes; 18S and 5.8S rRNAs are omitted for simplicity. Black circles represent unviable spores, while the blue/red circles rare viable spore. Outcrossing followed by nuclear fusion (1) or incomplete lineage sorting (2) give rise to divergent repeats in individual genomes, and set evolutionary processes in motion leading to different patterns of intra-genomic variation. A polymorphic/hybrid ancestor can rarely produce viable meiotic spores with chimeric rDNA arrays (category I). Over time some lineages partially homogenize rDNA arrays (categories II and III), whereas other descendants can retain both ribosomal variants due to low levels of homogenisation by recombination (category IV). Abbreviation: ILS: incomplete lineage sorting. Alternatively, outcrossing between two divergent haploid cells results in a transient heterokaryon (without nuclear fusion) (3) which undergoes introgression, i.e. the transfer of genetic materials from a donor to a recipient cell. The blue rDNA array and the introgressed red array donated by the pink parental cell recombine, leading to chimeric rDNAs (category I).

Hybridization can occur when the process leading to the extent evolutionary lineages does not consist of rapid speciation followed by immediate and complete genetic isolation [65]. In *Saccharomyces*, synthetic interspecific hybrids have been described to maintain both parental rDNA arrays [66–80]. Differently from synthetic hybrids, natural hybrids species *Saccharomyces pastorianus* [71], *Millerozyma* (*Pichia*) *farinosa* [72] and *Pichia sorbitophila* [73] exhibit unidirectional homogenization of rDNA repeats due to the loss of one parental rDNA array. In the *Z*. *rouxii* species complex, we did not observe the unidirectional dominance of one parental rDNA array, as reported for other wild hybrid species. Instead, bi-directional concerted evolution took place with four puzzling possible outcomes of lack of homogenization (category IV), partial homogenization (category II and III) and complete homogenization generating mosaic rDNA types (category I) (Fig 7). These alternative evolutionary possibilities imply that the rate of concerted evolution following putative allodiploidization events is variable in different regions of ribosomal tandem repeats, and that the extent of rDNA sequence homogenization varies among different individuals.

Two divergent rDNA arrays are expected in diploid yeast hybrids immediately after the merger of two parental genomes. Even if some patterns of rDNA sequence homogenization are consistent with recent interbreeding between two divergent parental populations, this hypothesis is not fully supported by the retention of multiple intra-individual haplotypes in rRNA coding regions of some strains (NBRC 10669 and NBRC 0505), as well as by the supposed haploid status of others (NBRC 10670 and NBRC 10672). Therefore, incomplete lineage sorting (ILS) may also be a plausible hypothesis alternative to hybridization. ILS of mixed rDNA arrays occurs when a common polymorphic ancestor that has two or more alleles (that is, haplotypes) at the rDNA locus divides into two lineages [74] (Fig 7). These alleles can be retained in the descendant branches, and when one of these lineages divides again, all three species lineages may carry all the ribosomal variants and, over time, homogenize the variants differently.

### Forces shaping contrasting patterns of homogenization

Regardless the origins of ribosomal heterogeneity (hybridization or ILS), it is assumed that, as time passes, recombination processes, such as unequal crossing over and gene conversion, continue to gradually homogenize individual unit sequences. However, the contrasting patterns of intra-individual variation uncovered here indicate that the time factor does not seem to be the only player in homogenizing divergent rDNAs, but other forces can shape the extent and direction of rDNA concerted evolution. In hybrids, spatial separation of rDNA arrays on non-homologous or homeologous chromosomes limits concerted evolution, as the homogenization of rDNA repeats is more effective within than between loci [9]. When intra-chromosomal homogenization exceeds inter-chromosomal homogenization, the dynamics of homogenization are analogous to the effects of gene flow: low levels of homogenization between different chromosomal loci would allow the accumulation of different ribotypes. Additionally, defects in recombination between homologous rDNA units hamper homogenization and give rise to incomplete concerted evolution [18]. Similarly, asexual propagation slows down rDNA sequence homogenization [75], whereas sexual reproduction and introgression may accelerate the rapid sorting of chimeric rDNA arrays from a polymorphic ancestor. The *Z*. *rouxii* species complex encompasses both asexual and sexual populations which could undergo different homogenization rates [35, 76]. For category I, we can speculate that homogenized chimeric rDNA arrays sorted after meiosis, when putative hybrid ancestors produced rare viable spores with recombinant rDNA-containing chromosomes (Fig 7). Alternatively, chimeric rDNAs could be interpreted as traces of introgression leading to rapid homogenization of chimeric rDNA sequences following outcrossing (Fig 7). One introgression mechanism proposed for accelerating segregation of aneuploids after syngamy is the unidirectional transfer of chromosomal segments from the donor to the recipient cells in newly formed zygotes prior to karyogamy (as reviewed by [65]). This is consistent with the haploid life cycle of *Zygosaccharomyces* yeasts, that frequently form transient heterokaryons after mating between haploid cells without nuclear fusion ([31] and references therein) (Fig 7).

In plants, epigenetic silencing of rDNA loci also influences evolutionary patterns of rDNA homogenization, because silenced arrays are less prone to concerted evolution than those actively transcribed [76]. Given that rRNA genes are highly transcribed and tightly controlled, any genomic modifications at rRNA loci (such as differential upregulation/suppression of rRNA variants) will translate into “extra-coding functions” affecting a multitude of cellular processes [78]. Divergent variants could have different levels of transcription, resulting in RNA molecules less diverse than the genes of the rDNA array [4]. Based on this observation, Sipiczki et al. [62] interpreted rDNA heterogeneity in D1/D2 domains as a signal of ineffective homogenization, which took place in fungi growing under selective pressure. Although the biological consequences of rDNA heterogeneity are largely unknown, there could be some selective advantages to evolve and maintain a variety of rDNA repeat sequences. For the prokaryotes having heterogeneous 16S rRNA genes, it has been hypothesized that multiple RNA operons are functionally differentiated and contribute to coping with a variety of environmental conditions [79, 80]. Interestingly, the majority of yeasts with intra-genomic ribosomal polymorphisms inhabit hostile environments. We speculate that the partial homogenization of coding and non-coding regions observed in categories II and III may give *Zygosaccharomyces* yeasts some functional advantages in growing under stress.

Taking all these arguments into account, rDNA evolution in the *Z*. *rouxii* complex could result from an intricate interplay of factors: 1) the evolutionary time which concerted evolution requires to homogenize divergent rDNA arrays, which is in turn affected by mode of reproduction; 2) the spatial organization of divergent rDNA arrays which can lie on the same chromosome or on non-homologous/homeologous chromosomes; 3) the functional constraints that shape the extent of rDNA sequence homogenization by epigenetic silencing mechanisms.

### Implications for yeast barcoding and reticulation detection

Both ILS and allodiploidization produce discrepancies between the gene-level phylogenetic tree and the overall species-level phylogenetic tree, because the phylogenetic tree for each ribosomal segment may or may not match the branching order for the species-level evolutionary tree. Discrepancies between gene- and species-level phylogenies introduce substantial challenges for the DNA barcode-based analysis of yeast biodiversity. One of the goals of modern taxonomy is to find a single easily PCR-amplifiable barcode identifier to diagnose taxa, increasing the speed, objectivity, and efficiency of species identification [81, 82]. In yeast taxonomy, ITS regions are preferred to D1/D2 domains, due to their higher discriminating power [83]. However, the setting of a cut-off delimiting within- and between-species divergence implies the existence of a barcoding gap, i.e. the difference between the highest intraspecific variation and the smallest interspecific divergence in a given dataset [83]. The rDNA barcoding becomes effective when there is no overlap between intraspecific variation and the interspecific divergence, and a threshold value for the species delimitation can thereby be established. The presence of several ribotypes within an individual shortens the barcoding gap and should be taken into consideration before using ribosomal markers to circumscribe yeast taxa [26]. In the *Z*. *rouxii* complex the extent of intra-individual ribosomal polymorphisms may invalidate rDNA-based species delimitation, but can be useful to elucidate reticulate evolutionary histories and infer ancestry or parental origins. Differently from other cryptic species, the *Z*. *rouxii* complex did not undergo complete loss of one or other divergent arrays and therefore has retained stronger phylogenetic signals for reconstructing reticulation history.

## Conclusions

The present study documents how yeasts of the *Z*. *rouxii* complex exhibit an unusually high level of intra-genomic rDNA variation. We demonstrate that diverse patterns of rDNA homogenization took place in individual genomes of these yeasts, including maintenance, partial or complete homogenization of divergent arrays. Our findings also highlight how concerted evolution does not act equally on all the regions of the ribosomal array, but homogenizes some portions ad retains polymorphisms in others. Our study indicates that the process of rDNA evolution is complicated in *Z*. *rouxii* species complex, and that no firm conclusion can be drawn on the direction of repeat homogenization. The resulting puzzling rDNA rearrangements reflect the dynamic nature and evolutionary genomic plasticity of this clade that frequently experienced gene duplication, ploidy change, aneuploidy and karyotyping variability. Furthermore, bi-directional concerted evolution of rDNA arrays suggests that structural (rDNA arrays located within or between chromosomes), temporal (timescale of ILS or hybridization events) and functional (epigenetic silencing) constraints contribute to make these multiple rDNA outcomes possible within a single clade. To the best of our knowledge, our study has provided the first evidence on the complexity of rDNA evolution in the *Z*. *rouxii* species complex.

## Supporting Information

S1 File Alignment of ITS1 sequences surveyed by direct sequencing and cloning. (RTF)

S2 File Alignment of ITS2 sequences surveyed by direct sequencing and cloning. (RTF)

S3 File Alignment of VR1 sequences surveyed by direct sequencing and cloning. (TXT)

S4 File Alignment of VR2 sequences surveyed by direct sequencing and cloning. (TXT)

S1 Table Details of yeast strains used in this study. (DOCX)

S2 Table Accession numbers of DNA sequences obtained in this study (DOC)

S3 Table Cloning libraries obtained for strains showing ITS heterogeneity. The minus symbols indicate inapplicable results; asterisks mark *in silico* digestion profiles. *Hae*III-profile of *Z*. *sapae* ITS haplotypes 1 to 3 are according to Solieri et al. [37].
(DOC)

S4 Table Estimate of evolutionary divergences between ITS sequences within and between strains. For the evolutionary divergence calculation, the intra-genomic ITS variants within a strain were compared to each other and to their closest relative, such as *Z*. *rouxii* (AM943655), strain CBS 4837 ITS copy 2 (HE664090), *Z*. *sapae* ITS copies 1 (AM279465) and 2 (AM2794964). All positions containing gaps and missing data were eliminated. Evolutionary analyses were conducted in MEGA6. Abbreviation: cp, copy.
(DOCX)

S5 Table Cloning libraries obtained for strains showing heterogeneity in 26S rDNA D1/D2 domains. The minus symbols indicate inapplicable results; asterisks mark *in silico* digestion profiles. Abbreviation: cp, copy.
(DOCX)

S6 Table Estimate of evolutionary divergences between D1/D2 sequences within and between strains. For the evolutionary divergence calculation, the intra-genomic D1/D2 sequences within a strain are compared to each other and to their closest relative, such as *Z*. *mellis* (U72164), *Z*. *sapae* (AJ966342) and *Z*. *rouxii* (AM943655). All positions containing gaps and missing data were eliminated. Evolutionary analyses were conducted in MEGA6. Abbreviation: cp, copy.
(DOCX)
