# Supplementary material for: Contrasting Patterns of rDNA Homogenization within the Zygosaccharomyces rouxii Species Complex
Source: PLoS One. 2016 Aug 8;11(8):e0160744. doi: 10.1371/journal.pone.0160744 (PMC4976873; doi:10.1371/journal.pone.0160744)
Supplement: S1 File — (RTF) [file pone.0160744.s001.rtf]

#MEGA
!Title allITS1_womellis;
!Format
   DataType=Nucleotide
   NSeqs=29 NSites=237
   Identical=. Missing=? Indel=-;


NBRC0495 cp1           TAGAAAATGA ----AAATCT CGA-AGAGCT --------GG GGGGGGGA-- AGAGCCTGCG
NBRC0495 cp2           TAGAAAATGA ----AAATCT CGA-AGAGCT --------GG GGGGGGGA-- AGAGCCTGCG
NBRC0505               TAGAAAATGA ----AAATCT CGA-AGAGCT --------GG GGGGGGGA-- AGAGCCTGCG
NBRC0845               TAGAAAATGA CGTGAACTCT TAACGGAGTT CTCTCAAAGT GTTGGAGGGG AAGGCCTGCG
NBRC10652 cp1          TAGAAAATGA CGTGAACTCT TAACGGAGTT CTCTCAAAGT GTTGGAGGGG AAGGCCTGCG
NBRC10652 cp2          TAGAAAATGA CGTGAACTCT TAACGGAGTT CTCTCAAAGT GTTGGAGGGG AAGGCCTGCG
NBRC10668              TAGAAAATGA CGTGAACTCT TAACGGAGTT CTCTCAAAGT GTTGGAGGGG AAGGCCTGCG
NBRC10669 cp1          TAGAAAATGA CGTGAACTCT TAACGGAGTT CTCTCAAAGT GTTGGAGGGG AAGGCCTGCG
NBRC10669 cp2          TAGAAAATGA ----AAATCT CGA-AGAGCT --------GG GGGGGGGG-- AGAGCCTGCG
NBRC10670 cp1          TAGAAAATGA CGTGAACTCT TAACGGAGTT CTCTCAAAGT GTTGGAGGGG AAGGCCTGCG
NBRC10670 cp2          TAGAAAATGA ----AAATCT CGA-AGAGCT --------GG GGGGGGG--- AGAGCCTGCG
NBRC10672 cp1          TAGAAAATGA CGTGAACTCT TAACGGAGTT CTCTCAAAGT GTTGGAGGGG AAGGCCTGCG
NBRC10672 cp2          TAGAAAATGA CGTGAACTCT TAACGGAGTT CTCTCAAAGT GTTGGAGGGG AAGGCCTGCG
M21 cp1                TAGAAAATGA CGTGAACTCT TAACGGAGTT CTCTCAAAGT GTTGGAGGGG AAGGCCTGCG
M21 cp2                TAGAAAATGA ----AAATCT CGA-AGAGCT --------GG GGGGGGGG-G AGAGCCTGCG
NBRC0525               TAGAAAATGA ----AAATCT CGA-AGAGCT --------GG GGGGGGGG-A AGAGCCTGCG
NCYC3042 (HE984156)    TAGAAAATGA ----AAATCT CGA-AGAGCT --------GG GGGGGGGGAG AGAGCCTGCG
Z.sapae cp2 (AM279464) TAGAAAATGA ----AAATCT CGA-AGAGCT --------GG GGGGGGGG-- AGAGCCTGCG
Z.sapae cp3 (AM279696) TAGAAAATGA ----AAATCT CGA-AGAGCT --------GG GGGGGGGG-G AGAGCCTGCG
Z.sapae cp1 (AM279465) TAGAAAATGA CGTGAACTCT TAACGGAGTT CTCTCAAAGT GTTGGAGGGG AAGGCCTGCG
CBS4837 cp2 (HE664090) TAGAAAATGA CGTGAACTCT TAACGGAGTT CTCTCAAAGT GTTGGAGGGG AAGGCCTGCG
CBS4837 cp1 (HE664088) TAGAAAATGA CGTGAACTCT TAACGGAGTT CTCTCAAAGT GTTGGAGGGG AAGGCCTGCG
CBS4837 cp3 (HE664089) TAGAAAATGA ----AAATCT CGA-AGAGCT --------GG GGGGGGG--- AGAGCCTGCG
CBS4838 cp2 (HE664091) TAGAAAATGA CGTGAACTCT TAACGGAGTT CTCTCAAAGT GTTGGAGGGG AAGGCCTGCG
CBS4838 cp3 (HE664092) TAGAAAATGA ----AAATCT CGA-AGAGCT --------GG GGGGGGG--- AGAGCCTGCG
CBS4838 cp1 (HE664093) TAGAAAATGA CGTGAACTCT TAACGGAGTT CTCTCAAAGT GTTGGAGGGG AAGGCCTGCG
ATCC42981_p(AM943657)  TAGAAAATGA ----AAATCT CGA-AGAGCT --------GG GGGGGGGA-- AGAGCCTGCG
ATCC42981_t(AM943656)  TAGAAAATGA CGTGAACTCT TAACGGAGTT CTCTCAAAGT GTTGGAGGGG AAGGCCTGCG
Z.rouxii (AM279465)    TAGAAAATGA CGTGAACTCT TAACGGAGTT CTCTCAAAGT GTTGGAGGGG AAGGCCTGCG

NBRC0495 cp1           CTTAATTGCG CGGCTTGATT TA---CCCTC CGCCTTTGAT ACACACAGTG GAGTTTCTGC
NBRC0495 cp2           CTTAATTGCG CGGCTTGATT TA---CCCTC CGCCTTTGAT ACACACAGTG GAGTTTCTGC
NBRC0505               CTTAATTGCG CGGCTTGATT TA---CCCTC CGCCTTTGAT ACACACAGTG GAGTTTCTGC
NBRC0845               CTTAATTGCG CGGCTGTTTT TAATCTCCTC CGCCTTTGAT ACACACATTG GAGTTTCTAC
NBRC10652 cp1          CTTAATTGCG CGGCTGTTTT TAATCTCCTC CGCCTTTGAT ACACACATTG GAGTTTCTAC
NBRC10652 cp2          CTTAATTGCG CGGCTGTTTT TAATCTCCTC CGCCTTTGAT ACACACATTG GAGTTTCTAC
NBRC10668              CTTAATTGCG CGGCTGTTTT TAATCTCCTC CGCCTTTGAT ACACACATTG GAGTTTCTAC
NBRC10669 cp1          CTTAATTGCG CGGCTGTTTT TAATCTCCTC CGCCTTTGAT ACACACATTG GAGTTTCTAC
NBRC10669 cp2          CTTAATTGCG CGGCTTGATT TA---CCCTC CGCCTTTGAT ACACACAGTG GAGTTTCTGC
NBRC10670 cp1          CTTAATTGCG CGGCTGTTTT TAATCTCCTC CGCCTTTGAT ACACACATTG GAGTTTCTAC
NBRC10670 cp2          CTTAATTGCG CGGCTTGATT TA---CCCTC CGCCTTTGAT ACACACAGTG GAGTTTCTGC
NBRC10672 cp1          CTTAATTGCG CGGCTGTTTT TAATCTCCTC CGCCTTTGAT ACACACATTG GAGTTTCTAC
NBRC10672 cp2          CTTAATTGCG CGGCTGTTTT TAATCTCCTC CGCCTTTGAT ACACACATTG GAGTTTCTAC
M21 cp1                CTTAATTGCG CGGCTGTTTT TAATCTCCTC CGCCTTTGAT ACACACATTG GAGTTTCTAC
M21 cp2                CTTAATTGCG CGGCTTGATT TA---CCCTC CGCCTTTGAT ACACACAGTG GAGTTTCTAC
NBRC0525               CTTAATTGCG CGGCTTGATT TA---CCCTC CGCCTTTGAT ACACACAGTG GAGTTTCTGC
NCYC3042 (HE984156)    CTTAATTGCG CGGCTTGATT TA---CCCTC CGCCTTTGAT ACACACAGTG GAGTTTCTAC
Z.sapae cp2 (AM279464) CTTAATTGCG CGGCTTGATT TA---CCCTC CGCCTTTGAT ACACACAGTG GAGTTTCTAC
Z.sapae cp3 (AM279696) CTTAATTGCG CGGCTTGATT TA---CCCTC CGCCTTTGAT ACACACAGTG GAGTTTCTAC
Z.sapae cp1 (AM279465) CTTAATTGCG CGGCTGTTTT TAATCTCCTC CGCCTTTGAT ACACACATTG GAGTTTCTAC
CBS4837 cp2 (HE664090) CTTAATTGCG CGGCTGTTTT TAATCTCCTC CGCCTTTGAT ACACACATTG GAGTTTCTAC
CBS4837 cp1 (HE664088) CTTAATTGCG CGGCTTGATT TA---CCCTC CGTCTTTGAT ACACACAGTG GAGTTTCTGC
CBS4837 cp3 (HE664089) CTTAATTGCG CGGCTTGATT TA---CCCTC CGCCTTTGAT ACACACAGTG GAGTTTCTGC
CBS4838 cp2 (HE664091) CTTAATTGCG CGGCTGTTTT TAATCTCCTC CGCCTTTGAT ACACACATTG GAGTTTCTAC
CBS4838 cp3 (HE664092) CTTAATTGCG CGGCTTGATT TA---CCCTC CGCCTTTGAT ACACACAGTG GAGTTTCTGC
CBS4838 cp1 (HE664093) CTTAATTGCG CGGCTGTTTT TAATCTCCTC CGCCTTTGAT ACACACATTG GAGTTTCTAC
ATCC42981_p(AM943657)  CTTAATTGCG CGGCTTGATT TA---CCCTC CGCCTTTGAT ACACACAGTG GAGTTTCTGC
ATCC42981_t(AM943656)  CTTAATTGCG CGGCTGTTTT TAATCTCCTC CGCCTTTGAT ACACACATTG GAGTTTCTAC
Z.rouxii (AM279465)    CTTAATTGCG CGGCTGTTTT TAATCTCCTC CGCCTTTGAT ACACACATTG GAGTTTCTAC

NBRC0495 cp1           TTTTTTGTTC TCTTTGGGGA AGTGCTTTTA AAGGCGTCTG TCCCCAGAGG TAAACACAAA
NBRC0495 cp2           TTTTTTGTTC TCTTTGGGGA AGTGCTTTTA AAGGCGTCTG TCCCCAGAGG TAAACACAAA
NBRC0505               TTTTTTGTTC TCTTTGGGGA AGTGCTTTTA AAGGCGTCTG TCCCCAGAGG TAAACACAAA
NBRC0845               TTTTTTGTTC TCTTTGGGAG GGTTCTGCT- ---------- CTCCCAGAGG TAAACACAAA
NBRC10652 cp1          TTTTTTGTTC TCTTTGGGAG GGTTCTGCT- ---------- CTCCCAGAGG TAAACACAAA
NBRC10652 cp2          TTTTTTGTTC TCTTTGGGAG GGTTCTGCT- ---------- CTCCCAGAGG TAAACACAAA
NBRC10668              TTTTTTGTTC TCTTTGGGAG GGTTCTGCT- ---------- CTCCCAGAGG TAAACACAAA
NBRC10669 cp1          TTTTTTGTTC TCTTTGGGAG GGTTCTGCT- ---------- CTCCCAGAGG TAAACACAAA
NBRC10669 cp2          TTTTTTGTTC TCTTTGGGGA AGTGCTTTTA AAGGCGTCTG TCCCCAGAGG TAAACACAAA
NBRC10670 cp1          TTTTTTGTTC TCTTTGGGAG GGTTCTGCT- ---------- CTCCCAGAGG TAAACACAAA
NBRC10670 cp2          TTTTTTGTTC TCTTTGGGGA AGTGCTTTTA AAGGCGTCTG TCCCCAGAGG TAAACACAAA
NBRC10672 cp1          TTTTTTGTTC TCTTTGGGAG GGTTCTGCT- ---------- CTCCCAGAGG TAAACACAAA
NBRC10672 cp2          TTTTTTGTTC TCTTTGGGAG GGTTCTGCT- ---------- CTCCCAGAGG TAAACACAAA
M21 cp1                TTTTTTGTTC TCTTTGGGAG GGTTCTGCT- ---------- CTCCCAGAGG TAAACACAAA
M21 cp2                TTTTTTGTTC TCTTTAGGGA AGTGCTTTTA AAGGCGTCTG TCCCCAGAGG TAAACACAAA
NBRC0525               TTTTTTGTTC TCTTTGGGGA AGTGCTTTTA AAGGCGTCTG TCCCCAGAGG TAAACACAAA
NCYC3042 (HE984156)    TTTTTTGTTC TCTTTGGGGA AGTGCTTTTA AAGGCGTCTG TCCCCAGAGG TAAACACAAA
Z.sapae cp2(AM279464)  TTTTTTGTTC TCTTTGGGGA AGTGCTTTTA AAGGCGTCTG TCCCCAGAGG TAAACACAAA
Z.sapae cp3(AM279696)  TTTTTTGTTC TCTTTGGGGA AGTGCTTTTA AAGGCGTCTG TCCCCAGAGG TAAACACAAA
Z.sapae cp1(AM279465)  TTTTTTGTTC TCTTTGGGAG GGTTCTGCT- ---------- CTCCCAGAGG TAAACACAAA
CBS4837 cp2(HE664090)  TTTTTTGTTC TCTTTGGGAG GGTTCTGCT- ---------- CTCCCAGAGG TAAACACAAA
CBS4837 cp1(HE664088)  TTTTTTGTTC TCTTTGGGGA AGTGCTTTTA AAGGCGTCTG TCCCCAGAGG TAAACACAAA
CBS4837 cp3(HE664089)  TTTTTTGTTC TCTTTGGGGA AGTGCTTTTA AAGGCGTCTG TCCCCAGAGG TAAACACAAA
CBS4838 cp2(HE664091)  TTTTTTGTTC TCTTTGGGAG GGTTCTGCT- ---------- CTCCCAGAGG TAAACACAAA
CBS4838 cp3(HE664092)  TTTTTTGTTC TCTTTGGGGA AGTGCTTTTA AAGGCGTCTG TCCCCAGAGG TAAACACAAA
CBS4838 cp1(HE664093)  TTTTTTGTTC TCTTTGGGAG GGTTCTGCT- ---------- CTCCCAGAGG TAAACACAAA
ATCC42981 p(AM943657)  TTTTTTGTTC TCTTTGGGGA AGTGCTTTTA AAGGCGTCTG TCCCCAGAGG TAAACACAAA
ATCC42981 t(AM943656)  TTTTTTGTTC TCTTTGGGAG GGTTCTGCT- ---------- CTCCCAGAGG TAAACACAAA
Z.rouxii (AM279465)    TTTTTTGTTC TCTTTGGGAG GGTTCTGCT- ---------- CTCCCAGAGG TAAACACAAA

NBRC0495 cp1           CAACATTTTT ATGAAATTAT AAAAAGTCAA AAACGAATT- --AAAACAAA ATATTCA
NBRC0495 cp2           CAACATTTTT ATGAAATTAT AAAAAGTCAA AAACGAATT- --AAAACAAA ATATTCA
NBRC0505               CAACATTTTT ATGAAATTAT AAAAAGTCAA AAACGAATT- --AAAACAAA ATATTCA
NBRC0845               CAAT-CTTTT ATTATACTAT TAACACAGTC AAATGAATTT TAAAAACAAA ATATTCA
NBRC10652 cp1          CAAT-CTTTT ATTATACTAT TAACACAGTC AAATGAATTT TAAAAACAAA ATATTCA
NBRC10652 cp2          CAAT-CTTTT ATTATACTAT TAACACAGTC AAATGAATTT TAAAAACAAA ATATTCA
NBRC10668              CAAT-CTTTT ATTATACTAT TAACACAGTC AAATGAATTT TAAAAACAAA ATATTCA
NBRC10669 cp1          CAAT-CTTTT ATTATACTAT TAACACAGTC AAATGAATTT TAAAAACAAA ATATTCA
NBRC10669 cp2          CAACATTTTT ATGAAATTAT AAAAAGTCAA AAACGAATT- --AAAACAAA ATATTCA
NBRC10670 cp1          CAAT-CTTTT ATTATACTAT TAACACAGTC AAATGAATTT TAAAAACAAA ATATTCA
NBRC10670 cp2          CAACATTTTT ATGAAATTAT AAAAAGTCAA AAACGAATT- --AAAACAAA ATATTCA
NBRC10672 cp1          CAAT-CTTTT ATTATACTAT TAACACAGTC AAATGAATTT TAAAAACAAA ATATTCA
NBRC10672 cp2          CAAT-CTTTT ATTATACTAT TAACACAGTC AAATGAATTT TAAAAACAAA ATATTCA
M21 cp1                CAAT-CTTTT ATTATACTAT TAACACAGTC AAATGAATTT TAAAAACAAA ATATTCA
M21 cp2                CAACATTTTT ATGAAATTAT AAAAAGTCAA AAACGAATT- --AAAACAAA ATATTCA
NBRC0525               CAACATTTTT ATGAAATTAT AAAAAGTCAA AAACGAATT- --AAAACAAA ATATTCA
NCYC3042 (HE984156)    CAACATTTTT ATGAAATTAT AAAAAGTCAA AAACGAATT- --AAAACAAA ATATTCA
Z.sapae cp2(AM279464)  CAACATTTTT ATGAAATTAT AAAAAGTCAA AAACGAATT- --AAAACAAA ATATTCA
Z.sapae cp3(AM279696)  CAACATTTTT ATGAAATTAT AAAAAGTCAA AAACGAATT- --AAAACAAA ATATTCA
Z.sapae cp1(AM279465)  CAAT-CTTTT ATTATACTAT TAACACAGTC AAATGAATTT TAAAAACAAA ATATTCA
CBS4837 cp2(HE664090)  CAAT-CTTTT ATTATACTAT TAACACAGTC AAATGAATTT TAAAAACAAA ATATTCA
CBS4837 cp1(HE664088)  CAACATTTTT ATGAAATTAT AAAAAGTCAA AAACGAATT- --AAAACAAA ATATTCA
CBS4837 cp3(HE664089)  CAACATTTTT ATGAAATTAT AAAAAGTCAA AAACGAATT- --AAAACAAA ATATTCA
CBS4838 cp2(HE664091)  CAAT-CTTTT ATTATACTAT TAACACAGTC AAATGAATTT TAAAAACAAA ATATTCA
CBS4838 cp3(HE664092)  CAACATTTTT ATGAAATTAT AAAAAGTCAA AAACGAATT- --AAAACAAA ATATTCA
CBS4838 cp1(HE664093)  CAAT-CTTTT ATTATACTAT TAACACAGTC AAATGAATTT TAAAAACAAA ATATTCA
ATCC42981 p(AM943657)  CAACATTTTT ATGAAATTAT AAAAAGTCAA AAACGAATT- --AAAACAAA ATATTCA
ATCC42981 t(AM943656)  CAAT-CTTTT ATTATACTAT TAACACAGTC AAATGAATTT TAAAAACAAA ATATTCA
Z.rouxii (AM279465)    CAAT-CTTTT ATTATACTAT TAACACAGTC AAATGAATTT TAAAAACAAA ATATTCA
