# Supplementary material for: Contrasting Patterns of rDNA Homogenization within the Zygosaccharomyces rouxii Species Complex
Source: PLoS One. 2016 Aug 8;11(8):e0160744. doi: 10.1371/journal.pone.0160744 (PMC4976873; doi:10.1371/journal.pone.0160744)
Supplement: S2 File — (RTF) [file pone.0160744.s002.rtf]

#MEGA
!Title fasta file;
!Format
   DataType=Nucleotide
   NSeqs=29 NSites=289
   Identical=. Missing=? Indel=-;


!Domain=Data;
#NBRC0495_ITS2_2_copy_1               TTCCCTCTCA AAC------- -----TTTAC GTTTGGTAGT GAGCGATACT CTACTCTGG-
#NBRC0495_ITS2_3_copy_2               TTCCCTCTCA AACATAGCTT TTATGTTTAT GTTTGGTAGT GAGCGATACT CT--TTTTG-
#NBRC0505_ITS2_3                      TTCCCTCTCA AATATAGCTT TTATGTTTAT GTTTGGTAGT GAGCGATACT CT--TTTTG-
#NBRC0845_ITS2_1                      TTCCCTCTCA AAC------- -----TTTAC GTTTGGTAGT GAGCGATACT CTACTCTGG-
#NBRC10652_ITS2_7_copy_1              TTCCCTCTCA AAC------- -----TTTAC GTTTGGTAGT GAGCGATACT CTACTCTGG-
#NBRC10652_ITS2_6_copy_2              TTCCCTCTCA AAC------- -----TTTAC GTTTGGTAGT GAGCGATACT CTACTCTGG-
#NBRC10668_ITS2_2                     TTCCCTCTCA AAC------- -----TTTAC GTTTGGTAGT GAGCGATACT CTACTCTGG-
#NBRC10669_ITS2_11_copy_1             TTCCCTCTCA AAC------- -----TTTAC GTTTGGTAGT GAGCGATACT CTACTCTGG-
#NBRC10669_ITS2_10_copy_2             TTCCCTCTCA AACATAGCTT TTATGTTTAT GTTTGGTAGT GAGCGATACT CT--TTTTG-
#NBRC10670_ITS2_6_copy_1              TTCCCTCTCA AAC------- -----TTTAC GTTTGGTAGT GAGCGATACT CTACTCTGG-
#NBRC10670_ITS2_7_copy_2              TTCCCTCTCA AAC------- -----TTTAC GTTTGGTAGT GAGCGATACT CTACTCTGG-
#10672_ITS2_4_copy_1                  TTCCCTCTCA AAC------- -----TTTAC GTTTGGTAGT GAGCGATACT CTACTCTGG-
#10672_ITS2_3copy2                    TTCCCTCTCA AAC------- -----TTTAC GTTTGGTAGT GAGCGATACT CTACTCTGG-
#M21_ITS2_1_copy_1                    TTCCCTCTCA AAC------- -----TTTAC GTTTGGTAGT GAGCGATACT CTACTCTGG-
#M21_ITS2_2_copy_2                    TTCCCTCTCA AACATAGCTT ------TTAT GTTTGGTAGT GAGCGATACT CT--TTTTG-
#NBRC0525_ITS2                        TTCCCTCTCA AACATAGCTT TTATGTTTAT GTTTGGTAGT GAGCGATACT CT--TTTTG-
#Zsapae_ABT301_copy_2_(AM279464)      TTCCCTCTCA AACATAGCTT ------TTAT GTTTGGTAGT GAGCGATACT CT--TTTTG-
#Zsapae_ABT301_copy_3_(AM279696)      TTCCCTCTCA AAC------- ----GCTTGC GTTTGGTAGT GAGCGATACT CTATCTGAGC
#Zsapae_ABT301_copy_1_(AM279465)      TTCCCTCTCA AAC------- -----TTTAC GTTTGGTAGT GAGCGATACT CTACTCTGG-
#CBS4837_copy2_(HE664090)             TTCCCTCTCA AAC------- -----TTTAC GTTTGGTAGT GAGCGATACT CTACTCTGG-
#CBS4837_copy1_(HE664088)             TTCCCTCTCA AAC------- -----TTTAC GTTTGGTAGT GAGCGATACT CTACTCTGG-
#CBS4837_copy_3_(HE664089)            TTCCCTCTCA AAC------- -----TTTAC GTTTGGTAGT GAGCGATACT CTACTCTGG-
#CBS4838_copy_2_(HE664091)            TTCCCTCTCA AAC------- -----TTTAC GTTTGGTAGT GAGCGATACT CTACTCTGG-
#CBS4838_copy_3_(HE664092)            TTCCCTCTCA AAC------- -----TTTAC GTTTGGTAGT GAGCGATACT CTACTCTGG-
#CBS4838_copy_1_(HE664093)            TTCCCTCTCA AAC------- -----TTTAC GTTTGGTAGT GAGCGATACT CTACTCTGG-
#ATCC42981_p-subgenome_(AM943657)     TTCCCTCTCA AACATAGCTT TTATGTTTAT GTTTGGTAGT GAGCGATACT CT--TTTTG-
#Rouxii(wolfe)_ITS_-5-3.seq           TTCCCTCTCA AAC------- -----TTTAC GTTTGGTAGT GAGCGATACT CTACTCTGG-
#NCYC3042_ITS2                        TTCCCTCTCA AACATAGCTT CTATGTTTAT GTTTGGTAGA GAGCGATACT CT--TTTTG-
#ATCC42981_t-subgenome_ITS2(AM943656) TTCCCTCTCA AAC------- -----TTTAC GTTTGGTAGT GAGCGATACT CTACTCTGG-

#NBRC0495_ITS2_2_copy_1               -AGTTTGCTT GAAAATGGGA GGCCATAG-G CGAAGCATTG CTTTCCAATC CTGCGGCCCT
#NBRC0495_ITS2_3_copy_2               -AGTTTGCTT GAAAGTGGGA GGCCATAG-G CGGAGCTTAG TTTGC----- ----------
#NBRC0505_ITS2_3                      -AGTTTGCTT GAAAGTGGGA GGCCATAG-G CGGAGCTTAG TTTGC----- ----------
#NBRC0845_ITS2_1                      -AGTTTGCTT GAAAATGGGA GGCCATAG-G CGAAGCATTG CTTTCCAATC CTGCGGCCCT
#NBRC10652_ITS2_7_copy_1              -AGTTTGCTT GAAAATGGGA GGCCATAG-G CGAAGCATTG CTTTCCAATC CTGCGGCCCT
#NBRC10652_ITS2_6_copy_2              -AGTTTGCTT GAAAATGGGA GGCCATAG-G CGGAGCTTAG TTTGC----- ----------
#NBRC10668_ITS2_2                     -AGTTTGCTT GAAAATGGGA GGCCATAG-G CGGAGCTTAG TTTGC----- ----------
#NBRC10669_ITS2_11_copy_1             -AGTTTGCTT GAAAATGGGA GGCCATAG-G CGGAGCTTAG TTTGC----- ----------
#NBRC10669_ITS2_10_copy_2             -AGTTTGCTT GAAAGTGGGA GGCCATAG-G CGGAGCTTAG TTTGC----- ----------
#NBRC10670_ITS2_6_copy_1              -AGTTTGCTT GAAAATGGGA GGCCATAG-G CGAAGCATTG CTTTCCAATC CTGCGGCCCT
#NBRC10670_ITS2_7_copy_2              -AGTTTGCTT GAAAATGGGA GGCCATAG-G CGAAGCATTG CTTTCCAATC CTGCGGCCCT
#10672_ITS2_4_copy_1                  -AGTTTGCTT GAAAATGGGA GGCCATAG-G CGAAGCATTG CTTTCCAATC CTGCGGCCCT
#10672_ITS2_3copy2                    -AGTTTGCTT GAAAATGGGA GGCCATAG-G CGGAGCTTAG TTTGC----- ----------
#M21_ITS2_1_copy_1                    -AGTTTGCTT GAAAATGGGA GGCCATAG-G CGAAGCATTG CTTTCCAATC CTGCGGCCCT
#M21_ITS2_2_copy_2                    -AGTTTGCTT GAAAGTGGGA GGCCATAG-G CGGAGCTTAG TTTGC----- ----------
#NBRC0525_ITS2                        -AGTTTGCTT GAAAGTGGGA GGCCATAG-G CGGAGCTTAG TTTGC----- ----------
#Zsapae_ABT301_copy_2_(AM279464)      -AGTTTGCTT GAAAGTGGGA GGCCATAG-G CGGAGCTTAG TTTGC----- ----------
#Zsapae_ABT301_copy_3_(AM279696)      TGACCCCCCC CGACCTGGGC GACTGGGGAG AGGGGAGCAG GAAGTGGAGT TTGCTTGAAA
#Zsapae_ABT301_copy_1_(AM279465)      -AGTTTGCTT GAAAATGGGA GGCCATAG-G CGAAGCATTG CTTTCCAATC CTGCGGCCCT
#CBS4837_copy2_(HE664090)             -AGTTTGCTT GAAAATGGGA GGCCATAG-G CGGAGCTTAG TTTGC----- ----------
#CBS4837_copy1_(HE664088)             -AGTTTGCTT GAAAATGGGA GGCCATAG-G CGAAGCATTG CTTTCCAATC CTGCGGCCCT
#CBS4837_copy_3_(HE664089)            -AGTTTGCTT GAAAATGGGA GGCCATAG-G CGAAGCATTG CTTTCCAATC CTGCGGCCCT
#CBS4838_copy_2_(HE664091)            -AGTTTGCTT GAAAATGGGA GGCCATAG-G CGGAGCTTAG TTTGC----- ----------
#CBS4838_copy_3_(HE664092)            -AGTTTGCTT GAAAATGGGA GGCCATAG-G CGAAGCATTG CTTTCCAATC CTGCGGCCCT
#CBS4838_copy_1_(HE664093)            -AGTTTGCTT GAAAATGGGA GGCCATAG-G CGAAGCATTG CTTTCCAATC CTGCGGCCCT
#ATCC42981_p-subgenome_(AM943657)     -AGTTTGCTT GAAAGTGGGA GGCCATAG-G CGGAGCTTAG TTTGC----- ----------
#Rouxii(wolfe)_ITS_-5-3.seq           -AGTTTGCTT GAAAATGGGA GGCCATAG-G CGAAGCATTG CTTTCCAATC CTGCGGCCCT
#NCYC3042_ITS2                        -AGTTTGCTT GAAAGTGGGA GGCCATAG-G CGGAGCTTAG TTTGC----- ----------
#ATCC42981_t-subgenome_ITS2(AM943656) -AGTTTGCTT GAAAATGGGA GGCCATAG-G CGAAGCATTG CTTTCCAATC CTGCGGCCCT

#NBRC0495_ITS2_2_copy_1               CTGCTTACTT CCCCTTGTGG GTTGTGGCAG GGG-AAAGCG GGAGG----- CGCCTTGCCA
#NBRC0495_ITS2_3_copy_2               ---------- ---------G ACTGTGCCGA GAG-GCA-TG GGAGC----- GGCCTAGCCA
#NBRC0505_ITS2_3                      ---------- ---------G ACTGTGCCGA GAG-GCA-TG GGAGC----- GGCCTAGCCA
#NBRC0845_ITS2_1                      CTGCTTACTT CCCCTTGTGG GTTGTGGCAG GGG-AAAGCG GGAGG----- CGCCTTGCCA
#NBRC10652_ITS2_7_copy_1              CTGCTTACTT CCCCTTGTGG GTTGTGGCAG GGG-AAAGCG GGAGG----- CGCCTTGCCA
#NBRC10652_ITS2_6_copy_2              ---------- ---------G ACTGTGCCGA GAG-GCA-TG GGAGC----- GGCCTAGCCA
#NBRC10668_ITS2_2                     ---------- ---------G ACTGTGCCGA GAG-GCA-TG GGAGC----- GGCCTAGCCA
#NBRC10669_ITS2_11_copy_1             ---------- ---------G ACTGTGCCGA GAG-GCA-TG GGAGC----- GGCCTAGCCA
#NBRC10669_ITS2_10_copy_2             ---------- ---------G ACTGTGCCGA GAG-GCA-TG GGAGC----- GGCCTAGCCA
#NBRC10670_ITS2_6_copy_1              CTGCTTACTT CCCCTTGTGG GTTGTGGCAG GGG-AAAGCG GGAGG----- CGCCTTGCCA
#NBRC10670_ITS2_7_copy_2              CTGCTTACTT CCCCTTGTGG GTTGTGGCAG GGG-AAAGCG GGAGG----- CGCCTTGCCA
#10672_ITS2_4_copy_1                  CTGCTTACTT CCCCTTGTGG GTTGTGGCAG GGG-AAAGCG GGAGG----- CGCCTTGCCA
#10672_ITS2_3copy2                    ---------- ---------G ACTGTGCCGA GAG-GCA-TG GGAGC----- GGCCTAGCCA
#M21_ITS2_1_copy_1                    CTGCTTACTT CCCCTTGTGG GTTGTGGCAG GGG-AAAGCG GGAGG----- CGCCTTGCCA
#M21_ITS2_2_copy_2                    ---------- ---------G ACTGTGCCGA GAG-GCA-TG GGAGC----- GGCCTAGCCA
#NBRC0525_ITS2                        ---------- ---------G ACTGTGCCGA GAG-GCA-TG GGAGC----- GGCCTAGCCA
#Zsapae_ABT301_copy_2_(AM279464)      ---------- ---------G ACTGTGCCGA GAG-GCA-TG GGAGC----- GGCCTAGCCA
#Zsapae_ABT301_copy_3_(AM279696)      GTGGGAGGCC ATAGACGGAG CTTATCTTGA GTGCGCAGTT GAAGCTGCGA CGCCTGGCCG
#Zsapae_ABT301_copy_1_(AM279465)      CTGCTTACTT CCCCTTGTGG GTTGTGGCAG GGG-AAAGCG GGAGG----- CGCCTTGCCA
#CBS4837_copy2_(HE664090)             ---------- ---------G ACTGTGCCGA GAG-GCA-TG GGAGC----- GGCCTAGCCA
#CBS4837_copy1_(HE664088)             CTGCTTACTT CCCCTTGTGG GTTGTGGCAG GGG-AAAGCG GGAGG----- CGCCTTGCCA
#CBS4837_copy_3_(HE664089)            CTGCTTACTT CCCCTTGTGG GTTGTGGCAG GGG-AAAGCG GGAGG----- CGCCTTGCCA
#CBS4838_copy_2_(HE664091)            ---------- ---------G ACTGTGCCGA GAG-GCA-TG GGAGC----- GGCCTAGCCA
#CBS4838_copy_3_(HE664092)            CTGCTTACTT CCCCTTGTGG GTTGTGGCAG GGG-AAAGCG GGAGG----- CGCCTTGCCA
#CBS4838_copy_1_(HE664093)            CTGCTTACTT CCCCTTGTGG GTTGTGGCAG GGG-AAAGCG GGAGG----- CGCCTTGCCA
#ATCC42981_p-subgenome_(AM943657)     ---------- ---------G ACTGTGCCGA GAG-GCA-TG GGAGC----- GGCCTAGCCA
#Rouxii(wolfe)_ITS_-5-3.seq           CTGCTTACTT CCCCTTGTGG GTTGTGGCAG GGG-AAAGCG GGAGG----- CGCCTTGCCA
#NCYC3042_ITS2                        ---------- ---------G ACTGTGCCGA GAG-GCA-TG GGAGC----- GGCCTAGCCA
#ATCC42981_t-subgenome_ITS2(AM943656) CTGCTTACTT CCCCTTGTGG GTTGTGGCAG GGG-AAAGCG GGAGG----- CGCCTTGCCA

#NBRC0495_ITS2_2_copy_1               CGATA----- GTCGTATTAG GTTTTACCGA CTCGGCGAA- -AGTGAAGAG GTTTGCTTTT
#NBRC0495_ITS2_3_copy_2               CGAAAA---- GTCGTATTAG GTTTTACCGA CTCGGCGGAA TAGTGGAGAG GTTTCTTTTT
#NBRC0505_ITS2_3                      CGAAAA---- GTCGTATTAG GTTTTACCGA CTCGGCGGAA TAGTGGAGAG GTTTCTTTTT
#NBRC0845_ITS2_1                      CGATA----- GTCGTATTAG GTTTTACCGA CTCGGCGAA- -AGTGAAGAG GTTTGCTTTT
#NBRC10652_ITS2_7_copy_1              CGATA----- GTCGTATTAG GTTTTACCGA CTCGGCGAA- -AGTGAAGAG GTTTGCTTTT
#NBRC10652_ITS2_6_copy_2              CGAAAA---- GTCGTATTAG GTTTTACCGA CTCGGCGGAA TAGTGGAGAG GTTTCTTTTT
#NBRC10668_ITS2_2                     CGAAAA---- GTCGTATTAG GTTTTACCGA CTCGGCGGAA TAGTGGAGAG GTTTCTTTTT
#NBRC10669_ITS2_11_copy_1             CGAAAA---- GTCGTATTAG GTTTTACCGA CTCGGCGGAA TAGTGGAGAG GTTTCTTTTT
#NBRC10669_ITS2_10_copy_2             CGAAAA---- GTCGTATTAG GTTTTACCGA CTCGGCGGAA TAGTGGAGAG GTTTCTTTTT
#NBRC10670_ITS2_6_copy_1              CGATA----- GTCGTATTAG GTTTTACCGA CTCGGCGAA- -AGTGAAGAG GTTTGCTTTT
#NBRC10670_ITS2_7_copy_2              CGATA----- GTCGTATTAG GTTTTACCGA CTCGGCGAA- -AGTGAAGAG GTTTGCTTTT
#10672_ITS2_4_copy_1                  CGATA----- GTCGTATTAG GTTTTACCGA CTCGGCGAA- -AGTGAAGAG GTTTGCTTTT
#10672_ITS2_3copy2                    CGAAAA---- GTCGTATTAG GTTTTACCGA CTCGGCGGAA TAGTGGAGAG GTTTCTTTTT
#M21_ITS2_1_copy_1                    CGATA----- GTCGTATTAG GTTTTACCGA CTCGGCGAA- -AGTGAAGAG GTTTCTTTTT
#M21_ITS2_2_copy_2                    CGAAAA---- GTCGTATTAG GTTTTACCGA CTCGGCGGAA TAGTGGAGAG GTTTCTTTTT
#NBRC0525_ITS2                        CGAAAA---- GTCGTATTAG GTTTTACCGA CTCGGCGGAA TAGTGGAGAG GTTTCTTTTT
#Zsapae_ABT301_copy_2_(AM279464)      CGAAAA---- GTCGTATTAG GTTTTACCGA CTCGGCGGAA TAGTGGAGAG GTTTCTTTTT
#Zsapae_ABT301_copy_3_(AM279696)      CGAAAACGAA GTCGTATTAG GTCTTACCGA CTCGGCGAA- ---GGAAGTA GTGGACGGGG
#Zsapae_ABT301_copy_1_(AM279465)      CGATA----- GTCGTATTAG GTTTTACCGA CTCGGCGAA- -AGTGAAGAG GTTTGCTTTT
#CBS4837_copy2_(HE664090)             CGAAAA---- GTCGTATTAG GTTTTACCGA CTCGGCGGAA TAGTGGAGAG GTTTCTTTTT
#CBS4837_copy1_(HE664088)             CGATA----- GTCGTATTAG GTTTTACCGA CTCGGCGAA- -AGTGAAGAG GTTTGCTTTT
#CBS4837_copy_3_(HE664089)            CGATA----- GTCGTATTAG GTTTTACCGA CTCGGCGAA- -AGTGAAGAG GTTTGCTTTT
#CBS4838_copy_2_(HE664091)            CGAAAA---- GTCGTATTAG GTTTTACCGA CTCGGCGGAA TAGTGGAGAG GTTTCTTTTT
#CBS4838_copy_3_(HE664092)            CGATA----- GTCGTATTAG GTTTTACCGA CTCGGCGAA- -AGTGAAGAG GTTTGCTTTT
#CBS4838_copy_1_(HE664093)            CGATA----- GTCGTATTAG GTTTTACCGA CTCGGCGAA- -AGTGAAGAG GTTTGCTTTT
#ATCC42981_p-subgenome_(AM943657)     CGAAAA---- GTCGTATTAG GTTTTACCGA CTCGGCGGAA TAGTGGAGAG GTTTCTTTTT
#Rouxii(wolfe)_ITS_-5-3.seq           CGATA----- GTCGTATTAG GTTTTACCGA CTCGGCGAA- -AGTGAAGAG GTTTGCTTTT
#NCYC3042_ITS2                        CGAAAA---- GTCGTATTAG GTTTTACCGA CTCGGCGGAA TAGTGGAGAG GTTTCTTTTT
#ATCC42981_t-subgenome_ITS2(AM943656) CGATA----- GTCGTATTAG GTTTTACCGA CTCGGCGAA- -AGTGAAGAG GTTTGCTTTT

#NBRC0495_ITS2_2_copy_1               T-----AAAA AGAAGCAGGC AGCGT-CTGG CTTGACA-AA ATTCTCAAA
#NBRC0495_ITS2_3_copy_2               TTTTT-ATTC TAAGGTAAGC --CGT-CTGG CTAGACA-AA ATTCTCAAA
#NBRC0505_ITS2_3                      TTTTT-ATTC TAAGGTAAGC --CGT-CTGG CTAGACA-AA ATTCTCAAA
#NBRC0845_ITS2_1                      T-----AAAA AGAAGCAGGC AGCGT-CTGG CTTGGCA-AA ATTCTCAAA
#NBRC10652_ITS2_7_copy_1              T-----AAAA AGAAGCAGGC AGCGT-CTG- -TTGACA-AA ATTCTCAAA
#NBRC10652_ITS2_6_copy_2              TTTTT-ATTC TAAGGTAAGC --CGT-CTGG CTAGACA-AA ATCCTCAAA
#NBRC10668_ITS2_2                     TTTTT-ATTC TAAGGTAAGC --CGT-CTGG CTAGACA-AA ATTCTCAAA
#NBRC10669_ITS2_11_copy_1             TTTTT-ATTC TAAGGTAAGC --CGT-CTGG CTAGACA-AA ATTCTCAAA
#NBRC10669_ITS2_10_copy_2             TTTTT-ATTC TAAGGTAAGC --CGT-CTGG CTAGACA-AA ATTCTCAAA
#NBRC10670_ITS2_6_copy_1              T-----AAAA AGAAGCAGGC AGCGT-CTGG CTTGACATAA ATTCTCAAA
#NBRC10670_ITS2_7_copy_2              T-----AAAA AGAAGCAGGC AGCGT-CTGG CTTGACA-AA ATTCTCAAA
#10672_ITS2_4_copy_1                  T-----AAAA AGAAGCAGGC AGCGT-CTGG CTTGACA-AA ATTCTCAAA
#10672_ITS2_3copy2                    TTTTT-ATTC TAAGGTAAGC --CGT-CTGG CTAGACA-AA ATTCTCAAA
#M21_ITS2_1_copy_1                    TTTTT-ATTC TAAGGTAAGC --CGT-CTGG CTAG------ ---------
#M21_ITS2_2_copy_2                    TTTTTTATTC TAAGGTAAGC --CGT-CTGG CTAGACA-AA ATTCTCAAA
#NBRC0525_ITS2                        TTTTT-ATTC TAAGGTAAGC --CGT-CTGG CTAGACA-AA ATTCTCAAA
#Zsapae_ABT301_copy_2_(AM279464)      TTTTT-ATTC TAAGGTAAGC --CGT-CTGG CTAGACA-AA ATTCTCAAA
#Zsapae_ABT301_copy_3_(AM279696)      GGAAAAGAGC AGAGCTCTTT TGCTTGCTGG CTTGACA-GA ATTCTCAAA
#Zsapae_ABT301_copy_1_(AM279465)      T-----AGGA GCAGGCAG-- --CGT-CTGG CTTGACA-AA ATTCTCAAA
#CBS4837_copy2_(HE664090)             TTTTT-ATTC TAAGGTAAGC --CGT-CTGG CTAGACA-AA ATTCTCAAA
#CBS4837_copy1_(HE664088)             T-----AAAA AGAAGCAGGC AGCGT-CTGG CTTGACA-AA ATTCTCAAA
#CBS4837_copy_3_(HE664089)            T-----AAAA AGAAGCAGGC AGCGT-CTGG CTTGACA-AA ATTCTCAAA
#CBS4838_copy_2_(HE664091)            TTTTT-ATTC TAAGGTAAGC --CGT-CTGG CTAGACA-AA ATTCTCAAA
#CBS4838_copy_3_(HE664092)            T-----AAAA AGAAGCAGGC AGCGT-CTGG CTTGACA-AA ATTCTCAAA
#CBS4838_copy_1_(HE664093)            T-----AAAA AGAAGCAGGC AGCGT-CTGG CTTGACA-AA ATTCTCAAA
#ATCC42981_p-subgenome_(AM943657)     TTTTT-ATTC TAAGGTAAGC --CGT-CTGG CTAGACA-AA ATTCTCAAA
#Rouxii(wolfe)_ITS_-5-3.seq           T-----AAAA AGAAGCAGGC AGCGT-CTGG CTTGACA-AA ATTCTCAAA
#NCYC3042_ITS2                        TTTTT-ATTC TAAGGTAAGC --CGT-CTGG CTAGACA-AA ATTCTCAAA
#ATCC42981_t-subgenome_ITS2(AM943656) T-----AAAA AGAAGCAGGC AGCGT-CTGG CTTGACA-AA ATTCTCAAA
