# Supplementary material for: Contrasting Patterns of rDNA Homogenization within the Zygosaccharomyces rouxii Species Complex
Source: PLoS One. 2016 Aug 8;11(8):e0160744. doi: 10.1371/journal.pone.0160744 (PMC4976873; doi:10.1371/journal.pone.0160744)
Supplement: S1 Table — (DOCX) [file pone.0160744.s005.docx]

**S1 Table. Details of yeast strains used in this study**

| **Taxonomical position** | **Strain** | **Other culture collections** | **Source** | **Country** | **Genetic features** |
| --- | --- | --- | --- | --- | --- |
| *Z. bailii* | CBS 680^T^ | ATCC 58445^T^;DBVPG 6287^T^, NCYC 1416^T^, NRRL Y-2227^T^ | Beer | Japan | haploid strain |
| *Z. mellis* | CBS 736^T^ | DBVPG 6476 ^T^; MUCL 39114 ^T^, NCYC 2403 ^T^, NRRL Y-7559 ^T^, NRRL Y-12628 ^T^ | Honey | USA | nr |
| *Z. rouxii* | CBS 732^T^ | ATCC 2623^T^, MUCL 30254^T^, NCYC 568^T^, NRRL Y-229^T^, NBRC 1623^T^ | concentrated grape must | Italy | haploid homothallic strain |
| *Z. sapae* | ABT301^T^ | CBS 12607T, MUCL 54092 | TBV | Italy | diploid strain; aααα genotype |
|  | ABT601 | CBS 12608, MUCL 54093 | TBV | Italy | diploid strain |
| *Z. pseudorouxii* | NCYC 3042 | CBS 9951 | Sugar | UK | nr |
| mosaic lineage | ATCC 42981 | JCM 22060 | Miso | Japan | allodiploid strain |
|  | CBS 4837 | NCYC 1682, NRRL Y-2547 | Miso | Japan | aneuploid heterothallic strain; a mating behaviour |
|  | CBS 4838 | NRRL Y-2548 | Miso | Japan | aneuploid heterothallic strain; α mating behaviour |
| *Z. rouxii*^a^ | NBRC 0495 | IFO 0495 | nr | Japan | nr |
|  | NBRC 0505 | IFO 0505 | shoyu yeast | Japan | nr |
|  | NBRC 0506 | IFO 0506 | shoyu yeast | Japan | α mating behaviour |
|  | NBRC 0521 | IFO 0521 | shoyu moromi | Japan | α mating behaviour |
|  | NBRC 0523 | IFO 0523 | shoyu moromi | Japan | α mating behaviour |
|  | NBRC 0525 | IFO 0525 | miso paste | Japan | nr |
|  | NBRC 0845 | IFO 0845 | soya mash | Japan | nr |
|  | NBRC 0846 | IFO 0846 | soya mash | Japan | α mating type |
|  | NBRC 10652 | ATCC 26390 | - | Japan | segregant of NRRL 2547(*MAT*a) x NRRL 2548(*MAT*α) hybrid; *MAT*a genotype; a mating behaviour |
|  | NBRC 10655 | ATCC 26398 | - | Japan | segregant of Lys-M5(*MAT*a) x Lys-M12 (*MAT*α) hybrid; *MAT*α/*MAT*α genotype; α mating behaviour |
|  | NBRC 10668 | IFO 10668 | shoyu mash | Japan | α mating type |
|  | NBRC 10669 | IFO 10669 | shoyu mash | Japan | a mating type |
|  | NBRC 10670 | nr | nr | nr | *MAT*α/*MAT*α genotype; α mating behaviour |
|  | NBRC 10672 | nr | shoyu yeast | Japan | *MAT*α/*MAT*α genotype; α mating behaviour |
| Novel isolates | M21, M22, M23, M25 | - | TBV sample 1 | Italy | - |
|  | B8911, B8932, B8933, B8941, B8943, B89221 | - | TBV sample 2 | Italy | - |
|  | 2, 3, 4, 7, 9, 12, 23, 24, 27, 35, 40, 41, 56, 68, 70, 76, | - | spoiled honey 1 | Italy | - |
|  | 1CF, 5CF, 6C | - | spoiled honey 2 | Italy | - |

^a^: taxonomical position assigned in NBRC database. Abbreviations: -, not applicable; nr, not reported; TBV; traditional balsamic vinegar.
