# Supplementary material for: Contrasting Patterns of rDNA Homogenization within the Zygosaccharomyces rouxii Species Complex
Source: PLoS One. 2016 Aug 8;11(8):e0160744. doi: 10.1371/journal.pone.0160744 (PMC4976873; doi:10.1371/journal.pone.0160744)
Supplement: S2 Table — (DOC) [file pone.0160744.s006.doc]

**S2 Table**. Accession numbers of DNA sequences obtained in this study

| **Phylogenetic marker** | **Strain ( intragenomic variant code)** | **Accession number** |
| --- | --- | --- |
| ITS regions | NBRC 0495 (copy 1) | LN849114 |
|  | NBRC 0495 (copy 2) | LN849115 |
|  | NBRC 10652 (copy 1) | LN849116 |
|  | NBRC 10652 (copy 2) | LN849117 |
|  | NBRC 10669 (copy 1) | LN849118 |
|  | NBRC 10669 (copy 2) | LN849119 |
|  | NBRC 10670 (copy 1) | LN849120 |
|  | NBRC 10670 (copy 2) | LN849121 |
|  | NBRC 10672 (copy 1) | LN849122 |
|  | NBRC 10672 (copy 2) | LN849123 |
|  | M21 (copy 1) | LN849124 |
|  | M21 (copy 2) | LN849125 |
|  | NBRC 0505 | LN849126 |
|  | NBRC 0525 | LN849127 |
|  | NBRC 0845 | LN849128 |
|  | NBRC 10668 | LN849129 |
|  | 2 | LN849130 |
|  | 4 | LN849131 |
|  | 70 | LN849132 |
|  | 5CF | LN849133 |
|  | B8911 | LN849134 |
|  | B8943 | LN849135 |
| 26S rDNA D1/D2 domains | NBRC 0495 (copy m) | LN849092 |
|  | NBRC 0495 (copy s) | LN849093 |
|  | NBRC 10652 (copy r) | LN849101 |
|  | NBRC 10652 (copy s) | LN849100 |
|  | NBRC 10669 (copy m) | LN849103 |
|  | NBRC 10669 (copy s) | LN849104 |
|  | NBRC 10669 (copy r) | LN849105 |
|  | NBRC 10669 (copy r*) | LN849106 |
|  | NBRC 10670 (copy r) | LN849108 |
|  | NBRC 10670 (copy s) | LN849107 |
|  | NBRC 10672 (copy r) | LN849110 |
|  | NBRC 10672 (copy s) | LN849111 |
|  | M21 | LN849109 |
|  | NBRC 0505 (copy m) | LN849094 |
|  | NBRC 0505 (copy s) | LN849095 |
|  | NBRC 0505 (copy r) | LN849096 |
|  | NBRC 0525 (copy r) | LN849098 |
|  | NBRC 0525 (copy s) | LN849097 |
|  | NBRC 0845 | LN849099 |
|  | NBRC 10668 | LN849102 |
|  | 2 | LN849086 |
|  | 4 | LN849087 |
|  | 70 | LN849091 |
|  | 5CF | LN849088 |
|  | 9 | LN849089 |
|  | 41 | LN849090 |
|  | B8911 | LN849112 |
|  | B8943 | LN849113 |
