# Supplementary material for: Contrasting Patterns of rDNA Homogenization within the Zygosaccharomyces rouxii Species Complex
Source: PLoS One. 2016 Aug 8;11(8):e0160744. doi: 10.1371/journal.pone.0160744 (PMC4976873; doi:10.1371/journal.pone.0160744)
Supplement: S3 Table — The minus symbols indicate inapplicable results; asterisks mark in silico digestion profiles. HaeIII-profile of Z. sapae ITS haplotypes 1 to 3 are according to Solieri et al. [37]. (DOC) [file pone.0160744.s007.doc]

**S3 Table**. **Cloning libraries obtained for strains showing ITS heterogeneity**. The minus symbols indicate inapplicable results; asterisks mark *in silico* digestion profiles. *Hae*III-profile of *Z. sapae* ITS haplotypes 1 to 3 are according to Solieri et al. [37].

| **Strains** | **Strain *Hae*III profile**  **(bp)** | **Clone identifiers** | **Clone *Hae*III profile** | **Designation** | **Blastn hits**  **(Accession number; identity percentage %)** |  |
| --- | --- | --- | --- | --- | --- | --- |
| **CBS 732^T^** | 390-210-90 | - | - | AM279465 | - |  |
| **CBS 736^T^** | 570-230 | - | - | AY046190 | - |  |
| **JCM16825^T^** | 390-200-110* | - | - | AB565768 | - |  |
| **NCYC 3042** | 480-170-70 | - | - | HE984156 |  |  |
| **ABT301^T^** | 510-480-390-250-210-170-90 | - | 390-210-90 | cp 1 (AM279465) | *Z. rouxii* (AM943655;99) |  |
|  |  | - | 480-170-70 | cp 2 (AM279464) | NCYC 3042 (HE984156;99) |  |
|  |  | - | 510-170-70 | cp 3 (AM279696) | NBRC 0485 (AB302839;95) |  |
| **ATCC 42981** | 480-390-250-210-170-90 | - | 390-210-90* | t-subgenome (AM943656) | *Z. sapae* cp 1 (AM279465;98) |  |
|  |  | - | 480-170* | p-subgenome (AM943657) | *Z. sapae* cp 2 (AM279464;99) |  |
| **NBRC 0495** | 480-440-220-170 | 1,2,6,7,8,10,13,14,15 | 480-220 | cp 1 | CBS 4838 cp 3 (HE664092;99) |  |
|  |  | 3,4,5,9,11,12 | 480-170-70 | cp 2 | *Z. sapae* cp 2 (AM279464;99) |  |
| **NBRC 10652** | 390-210-170-90 | 7,16 | 390-210-90 | cp 1 | *Z. rouxii* (AM943655;100) |  |
|  |  | 1,2,3,4,5,6,8,9,10,11,12,13,14,15 | 390-170-90 | cp 2 | CBS 4837 cp 2 (HE664090;99) |  |
| **NBRC 10669** | 480-390-190-90 | 7,11 | 390-190-90 | cp 1 | CBS 4838 cp 2 (HE664091;99) |  |
|  |  | 1,2,3,4,5,6,8,9,10,12,13,14,15 | 480-170-70 | cp 2 | *Z. sapae* cp 2 (AM279464;99) |  |
| **NBRC 10670** | 480-390-210-90 | 1,2,3,4,5,6,8,9,10,12,13,15 | 390-210-90 | cp 1 | *Z. rouxii* (AM943655;100) |  |
|  |  | 7,14 | 480-210 | cp 2 | CBS 4838 cp 3 (HE664092;99) |  |
| **NBRC 10672** | 480-390-250-210-170-90-70 | 4,11 | 390-210-90 | cp 1 | *Z. rouxii* (AM943655;100) |  |
|  |  | 1,2,3,5,6,7,8,9,10,12,13,14 | 390-190-90 | cp 2 | CBS 4837 cp2 (HE664090; 99) |  |
| **M21** | 510-480-390-250-210-170-90 | 1,3,4,5,6,7,8,12,14,15 | 390,210,90 | cp 1 | *Z. sapae* cp 1 (AM279465;98) |  |
|  |  | 2,9,10,11,13 | 480-170-70 | cp 2 | *Z. sapae* cp 2 (AM279464;99) | |
