# Supplementary material for: Contrasting Patterns of rDNA Homogenization within the Zygosaccharomyces rouxii Species Complex
Source: PLoS One. 2016 Aug 8;11(8):e0160744. doi: 10.1371/journal.pone.0160744 (PMC4976873; doi:10.1371/journal.pone.0160744)
Supplement: S4 Table — For the evolutionary divergence calculation, the intra-genomic ITS variants within a strain were compared to each other and to their closest relative, such as Z. rouxii (AM943655), strain CBS 4837 ITS copy 2 (HE664090), Z. sapae ITS copies 1 (AM279465) and 2 (AM2794964). All positions containing gaps and missing data were eliminated. Evolutionary analyses were conducted in MEGA6. Abbreviation: cp, copy. (DOCX) [file pone.0160744.s008.docx]

**S4 Table. Estimate of evolutionary divergences between ITS sequences within and between strains**. For the evolutionary divergence calculation, the intragenomic ITS variants within a strain were compared to each other and to their closest relative, such as *Z. rouxii* (AM943655), strain CBS 4837 ITS copy 2 (HE664090), *Z. sapae* ITS copies 1 (AM279465) and 2 (AM2794964). All positions containing gaps and missing data were eliminated. Evolutionary analyses were conducted in MEGA6. Abbreviation: cp, copy.

| **Strains with two ITS haplotype** | **Strain most similar to cp 1** | **Strains most similar to cp 2** | **Evolutionary divergence** | | |
| --- | --- | --- | --- | --- | --- |
|  |  |  | **cp 1 *vs* cp 2** | **cp 1 to nearest strain** | **cp 2 to nearest strain** |
| NBRC 0495 | CBS 4838 cp 3 | *Z. sapae* cp 2 | 0.066 | 0.002 | 0.005 |
| NBRC 10652 | *Z. rouxii* CBS 732^T^ | CBS 4837 cp 2 | 0.058 | 0.000 | 0.002 |
| NBRC 10669 | CBS 4837 cp 2 | *Z. sapae* ABT301^T^ cp 2 | 0.074 | 0.000 | 0.003 |
| NBRC 10670 | *Z. rouxii* CBS 732 ^T^ | CBS 4838 cp 3 | 0.077 | 0.000 | 0.000 |
| NBRC 10672 | *Z. rouxii* CBS 732 ^T^ | CBS 4837 cp 2 | 0.057 | 0.000 | 0.000 |
| M21 | *Z. sapae* ABT301^T^ cp 1 | *Z. sapae* ABT301^T^ cp 2 | 0.110 | 0.017 | 0.002 |
