# Supplementary material for: Contrasting Patterns of rDNA Homogenization within the Zygosaccharomyces rouxii Species Complex
Source: PLoS One. 2016 Aug 8;11(8):e0160744. doi: 10.1371/journal.pone.0160744 (PMC4976873; doi:10.1371/journal.pone.0160744)
Supplement: S5 Table — The minus symbols indicate inapplicable results; asterisks mark in silico digestion profiles. Abbreviation: cp, copy. (DOCX) [file pone.0160744.s009.docx]

**S4 Table**. **Cloning libraries obtained for strains showing 26S rDNA D1/D2 heterogeneity**. The minus symbols indicate not applicable results, whereas asterisks mark *in silico* digestion profiles.

| **Strains** | **Strain *Ava*I profile (bp)** | **Clone identifiers** | **Clone *Ava*I profile** | **Designation** | **Blastn hits**  **(Accession number; identity percentage %)** |
| --- | --- | --- | --- | --- | --- |
| **CBS 732^T^** | 510-100 | - | - | AM943655 | - |
| **CBS 736^T^** | 430-100 | - | - | U72164 | - |
| **NCYC 3042** | 390-100 | - | - | AJ555406 | - |
| **ABT301^T^** | 390-100 | - | - | AJ966342 | - |
| **ATCC 42981** | 510-70* | - | - | t-subgenome (AM943656) | - |
|  | 390-80* | - | - | p-subgenome (AM943657) | - |
| **NBRC 0495** | 510-390-100 | 1, 9, 10, 11, 13 | 510-100 | copy m | *Z. mellis* (AJ745090; 92) |
|  |  | 2, 3,4, 5, 6, 7, 8, 12, 14, 15 | 390-100 | copy s | *Z. sapae* (AJ966342; 99) |
| **NBRC 0505** | 510-430-390-100 | 12, 16 | 430-100 | copy m | *Z. mellis* (AJ745090; 99) |
|  |  | 1, 2, 3, 4, 5, 6, 7, 8, 9, 10, 11, 13, 14, 15 | 390-100 | copy s | *Z. sapae* (AJ966342; 99) |
|  |  | 8, 17 | 510-100 | copy r | *Z. rouxii* (AM943655; 99) |
| **NBRC 0525** | 510-390-100 | 3, 4, 6, 7, 12, 13, 15 | 390-100 | copy s | *Z. sapae* (AJ966342; 99) |
|  |  | 1, 2, 5, 8, 9, 10, 11, 14, 16 | 510-100 | copy r | *Z. rouxii* (AM943655; 99) |
| **NBRC 10652** | 510-390-100 | 1, 2, 3, 4, 5, 6, 7, 9, 10, 11, 15 | 390-100 | copy s | *Z. sapae* (AJ966342; 99) |
|  |  | 12, 13, 14 | 510-100 | copy r | *Z. rouxii* (U72163; 100) |
| **NBRC 10669** | 510-430-390-100 | 2, 12 | 430-100 | copy m | *Z. mellis* (AJ745090; 99) |
|  |  | 1, 4, 5, 8 | 390-100 | copy s | *Z. sapae* (AJ966342; 99) |
|  |  | 11, 16 | 430-100 | copy r* | *Z. rouxii* (AM943655; 99) |
|  |  | 3, 6, 7, 9, 10, 13, 14, 15 | 510-70 | copy r | *Z. rouxii* (AM943655; 100) |
| **NBRC 10670** | 510-390-100 | 2, 9 | 390-100 | copy s | *Z. sapae* (AJ966342; 99) |
|  |  | 1, 3, 4, 5, 6, 7, 10, 11, 12, 13, 14,15 | 510-100 | copy r | *Z. rouxii* (AM943655; 99) |
| **NBRC 10672** | 510-390-100 | 3, 4, 5, 6, 7, 10, 11, 13, 14 | 390-100 | copy s | *Z. sapae* (AJ966342; 100) |
|  |  | 1, 2, 8, 9, 12, 15 | 510-100 | copy r | *Z. rouxii* (AM943655; 100) |
