# Supplementary material for: Contrasting Patterns of rDNA Homogenization within the Zygosaccharomyces rouxii Species Complex
Source: PLoS One. 2016 Aug 8;11(8):e0160744. doi: 10.1371/journal.pone.0160744 (PMC4976873; doi:10.1371/journal.pone.0160744)
Supplement: S6 Table — For the evolutionary divergence calculation, the intra-genomic D1/D2 sequences within a strain are compared to each other and to their closest relative, such as Z. mellis (U72164), Z. sapae (AJ966342) and Z. rouxii (AM943655). All positions containing gaps and missing data were eliminated. Evolutionary analyses were conducted in MEGA6. Abbreviation: cp, copy. (DOCX) [file pone.0160744.s010.docx]

**S6 Table. Estimate of evolutionary divergences between D1/D2 sequences within and between strains.** For the evolutionary divergence calculation, the intragenomic D1/D2 sequences within a strain are compared to each other and to their closest relative, such as *Z. mellis* (U72164), *Z. sapae* (AJ966342) and *Z. rouxii* (AM943655). All positions containing gaps and missing data were eliminated. Evolutionary analyses were conducted in MEGA6. Abbreviation: cp, copy.

| **Strains with two D1/D2 variants** | | **Strain most similar to cp m/r** | | **Strain most similar to cp s** | | **Evolutionary divergence** | | | | | |  |  |  | | |  |  | | | |  |  |  |  |
| --- | --- | --- | --- | --- | --- | --- | --- | --- | --- | --- | --- | --- | --- | --- | --- | --- | --- | --- | --- | --- | --- | --- | --- | --- | --- |
|  |  |  |  |  |  | **cp m/r *vs.* cp s** | | **cp m/r to nearest strain** | | **cp s to nearest strain** | | | |  |  |  | | | |  |  | | | |  |
| NBRC 495 | | *Z. mellis* CBS 736^T^ | | *Z. sapae* ABT301^T^ | | 0.085 | | 0.032 | | 0.004 | | | |  |  |  | | | |  |  | | | |  |
| NBRC 0525 | | *Z. rouxii* CBS 732^T^ | | *Z. sapae* ABT301^T^ | | 0.026 | | 0.002 | | 0.000 | | | |  |  |  | | | |  |  | | | |  |
| NBRC 10652 | | *Z. rouxii* CBS 732^T^ | | *Z. sapae* ABT301^T^ | | 0.026 | | 0.000 | | 0.002 | | | |  |  |  | | | |  |  | | | |  |
| NBRC 10670 | | *Z. rouxii* CBS 732^T^ | | *Z. sapae* ABT301^T^ | | 0.035 | | 0.000 | | 0.010 | | | |  |  |  | | | |  |  | | | |  |
| NBRC 10672 | | *Z. rouxii* CBS 732^T^ | | *Z. sapae* ABT301^T^ | | 0.000 | | 0.000 | | 0.025 | | | |  |  |  | | | |  |  | | | |  |
|  | **Strain most similar to cp m** | | **Strain most similar to cp r** | | **Strain most similar to cp s** | |  | | **Evolutionary divergence** | | | | | | | | | | | | | |  | | |
| **Strains with more than two D1/D2 variants** |  |  |  |  |  |  | **inter-cp divergence*** | | | | **cp m to nearest strain** | | | **cp r to nearest strain** | | | | | **cp s to nearest strain** | | | |  |  | |
| NBRC 0505 | *Z. mellis* CBS 736^T^ | | *Z. rouxii* CBS 732^T^ | | *Z. sapae* ABT301^T^ | | 0.050/0.054/0.025 | | | | 0.005 | | | 0.002 | | | | | 0.002 | | | |  |  | |
| NBRC 10669^§^ | *Z. mellis* CBS 736^T^ | | *Z. rouxii* CBS 732^T^ | | *Z. sapae* ABT301^T^ | | 0.046/0.054/0.027 | | | | 0.002 | | | 0.002 | | | | | 0.004 | | | |  |  | |

*The inter-copy (cp) divergence is defined as evolutionary divergence between copies m and s, m and r, s and r, respectively. ^§^D1/D2 variant r* from strain NBRC 10669 showed an evolutionary divergence of 0.007 compared to variant r and was omitted from the analysis.
